# Gustatory avoidance of fatty acids by *Aedes aegypti* depends on an arthropod-specific TRP channel

**DOI:** 10.1073/pnas.2522818123

**Published:** 2026-02-09

**Authors:** Subash Dhakal, Angela E. Bontempo, Ramandeep Singh, Pratik Dhavan, Craig Montell

**Affiliations:** ^a^Neuroscience Research Institute, University of California, Santa Barbara, CA 93106; ^b^Department of Molecular, Cellular and Developmental Biology, University of California, Santa Barbara, CA 93106

**Keywords:** *Aedes aegypti*, *Drosophila melanogaster*, fatty acids, TRP channel, taste

## Abstract

Mosquito-disease vectors, such as *Aedes aegypti*, use their sense of taste before deciding whether to consume a blood meal, or fly away. However, the molecular mechanisms controlling gustatory decisions in mosquitoes are largely unknown. The transient receptor potential (TRP) channel, Painless1 (Pain1), is an intriguing candidate for participating in *Ae. aegypti* taste since *pain1* transcripts are detected in gustatory receptor neurons (GRNs). The *Drosophila* homolog, *painless* (*pain*) is also expressed in GRNs, where it is required for sensing allyl-isothiocyanate. Here, to identify additional gustatory roles for *pain* homologs, we first focused on *Drosophila pain*, which is widely expressed in multiple GRN classes. We demonstrated that *pain* mutations eliminated gustatory attraction to low fatty acids levels, repulsion to high levels, and fatty acid-induced action potentials. The attraction and repulsion depended on *pain* expression in different GRN classes. In contrast to *Drosophila*, when *Aedes* contacts fatty acids, they induce gustatory rejection only. *Aedes pain1* is expressed in taste organs, and is required for gustatory avoidance of fatty acids, and for fatty acid-induced action potentials. Given that Pain homologs are found in insects but not vertebrates, Pain1 represents an intriguing target for developing repellents to diminish biting, and transmission of infectious agents by mosquito disease vectors.

## Significance

The mosquito, *Aedes aegypti*, transmits the viruses afflicting millions of people with dengue and other diseases. Fatty acids (FAs) on skin can deter blood feeding. However, the sensors required for FA taste in mosquitoes are unknown. We identify the transient receptor potential channel Painless (Pain) as essential for FA taste in two dipterans. In *Drosophila*, Painless supports attraction to low doses and aversion to high doses of FAs via distinct gustatory receptor neurons (GRNs). In *Ae. aegypti*, the Pain1 ortholog is required for FA avoidance, which suppresses the decision to blood feed or nectar feed, and for FA-induced action potentials in GRNs. Because Pain homologs are arthropod-restricted, they offer a promising selective target for next-generation repellents.

Mosquito vectors spread the infectious agents that cause diseases afflicting hundreds of millions of people each year. *Aedes* (*Ae.*) *aegypti* are among the most concerning mosquitoes since their geographical range is increasing and because they infect people with the viruses that cause dengue, yellow fever, Zika, and other diseases (1). Only females spread these viruses since they require blood meals to obtain nutrients critical for egg development, and they often consume blood from more than one individual.

To navigate to their hosts, *Ae. aegypti* use multisensory integration (2–4). They home in from a long distance, ~1 to 15 m, by taking advantage of a repertoire of host-derived cues including exhaled CO_2_, the image of their target, and organic olfactory cues (1, 5). When they are within <0.8 m, they can then detect surface body temperature via thermal infrared (6). Once they are within a few centimeters, they sense the humidity and convection heat from the host (7, 8). Finally, when mosquitoes alight on a human, they sample the chemicals on skin before deciding to bite or fly away (9, 10). Therefore, understanding the molecular and cellular mechanisms through which mosquito disease vectors employ their sense of taste before withdrawing a blood meal is of great interest.

*Ae. aegypti* and other mosquitoes are endowed with taste organs distributed on multiple body parts (1, 9, 10). The first taste organs to contact a host are located on the terminal segments of the forelegs and midlegs, the tarsi. Surface chemicals are also sampled by the largest taste organ, the labellum, which consists of two bilaterally symmetrical lobes (palps) situated at the end of the proboscis. The tarsi and labella are decorated with taste sensilla that house gustatory receptor neurons (GRNs), which are used to identify both attractive and aversive chemicals on skin including fatty acids, ammonium, amino acids, salts, and metals (9, 11). Taste sensilla are also present near the ovipositor, which facilitate identifying favorable locations to lay eggs, and on the wing margins, although their functions on the mosquito’s wings have not been described. Currently, the receptors and channels in the tarsi and labella that contribute to evaluating the chemicals on skin are largely unknown, although a gustatory receptor (GR11) and an ENaC channel (Ppk301) have been implicated in detecting water in oviposition sites in *Ae. albopictu*s and *Ae. aegypti*, respectively (12, 13).

In contrast to our nascent understanding of the molecular components required for gustatory detection of host chemicals in mosquitoes, more than two decades of extensive studies in another dipteran, *Drosophila melanogaster*, has revealed a rich set of insect taste receptors and channels (14, 15). These include many members of two large families of so-called gustatory receptors (GRs), ionotropic receptors (IRs), and several Pickpockets. Each of these proteins are cation channels. In addition, multiple members of the transient receptor potential (TRP) family function in *Drosophila* taste (14, 15).

In *Ae. aegypti*, the TRP channels that are expressed at the highest levels in gustatory organs are TRPML and Painless (Pain) (16). However, *Aedes* TRPML is not a prime candidate for functioning in taste. In *Drosophila*, the TRPML is localized to lysosomes and endosomes of many cell types, and mutations in *trpml* compromise viability due to reduced autophagy (17). Currently, there is no evidence that *Drosophila* TRPML functions in gustation. In contrast, *Drosophila* Pain, which was originally identified based on its roles in thermal and mechanical nociception (18), is widely expressed in GRNs and contributes to avoiding allyl-isothiocyanate (AITC), the pungent component in wasabi (19, 20). However, Pain is not directly activated by AITC (21). *pain* mutant flies elicit normal gustatory aversion to quinine and high NaCl levels, and normal attraction to sugars (19) indicating that it does not have broad roles in taste.

To address the potential gustatory roles for Pain in *Ae. aegypti*, we first took advantage of the many existing genetic tools in *Drosophila* to investigate additional taste roles. On the basis of our findings in fruit flies, we then created genetic tools to characterize a *pain* homolog (*pain1*) in *Ae. aegypti*. We found that both *Drosophila* Pain and *Aedes* Pain1 have roles in fatty acid (FA) taste. While volatile FAs emanating from humans can be highly attractive to mosquitoes (22), contact with FAs is highly aversive to various mosquito disease vectors and suppresses biting and feeding (23–26).

In *Drosophila*, low concentrations of FAs are a nutrient source and are attractive (27–30), while high levels are toxic and aversive (31–33). *Drosophila* IRs, GRs, and a phospholipase C (NORPA) are involved in FA attraction (27–30, 34), while GRs contribute to FA aversion (32, 33).

Here, in support of previous findings on *Ae. aegypti* (23–26), we demonstrated that FAs elicited robust repulsion via gustation. We created mutations in *Ae. aegypti pain1*, as well as a gene reporter, which revealed that *pain1* is expressed in GRNs in both labella and tarsi. Using *pain1* knockout mosquitoes, we found that olfactory attraction to FAs was independent of *pain1*, while gustatory aversion to FAs depended on *pain1*. Consistent with the behavior, FA-induced action potentials were greatly reduced in taste sensilla from *pain1* mutants. These findings establish Pain channels as a molecular component facilitating FA taste detection across insect taxa—*Drosophila* and *Aedes*. Given that Pain homologs are specific to insects and subsets of other arthropods (35–38), and that *Ae. aegypti* Pain1 is necessary to signal the mosquitoes not to initiate feeding on an aversive class of chemical, we suggest that Pain provides an intriguing target for developing insect repellents to reduce the incidence of mosquito-borne disease.

## Results

### *Drosophila pain* Is Expressed across Subsets of all GRN Classes.

**Fig. 1.**
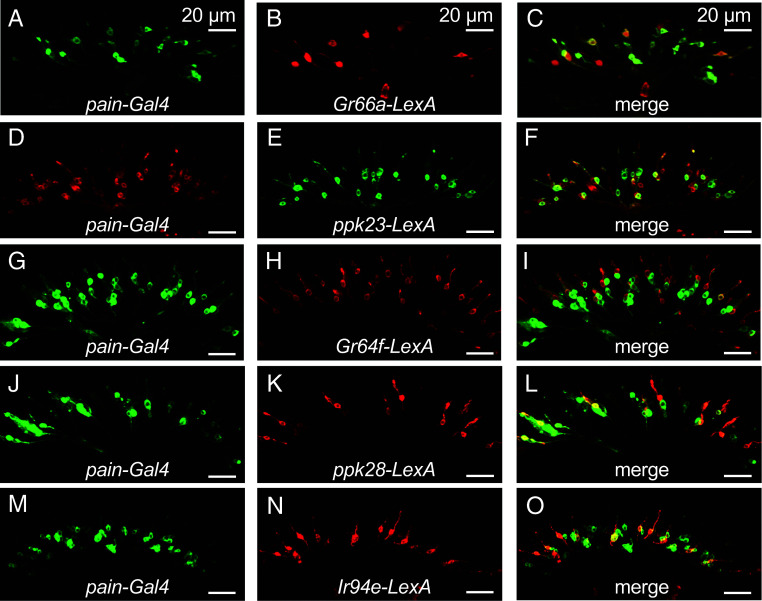
*Drosophila pain* is expressed in all labellar GRN classes. Each row indicates double-labeling using the *pain-Gal4* and the indicated GRN-specific driver. (*A*) *pain-Gal4* driving *40x UAS-IVS-mCD8::GFP.* (*B*) *Gr66a-LexA* driving *13x LexAop-6xmCherry.* (*C*) Merge of *A* and *B*. (*D*) *pain-Gal4* driving *UAS-10x-IVS- mCD8::RFP*. (*E*) *ppk23*-*LexA* driving *13x-LexAop2-mCD8::GFP.* (*F*) Merge of *D* and *E*. (*G*) *pain-Gal4* driving *40x UAS-IVS-mCD8::GFP*. (*H*) *Gr64f-LexA* driving *LexAop-rCD2::RFP* (*I*) Merge of *G* and *H*. (*J*) *pain-Gal4* driving *40x UAS-IVS-mCD8::GFP*. (*K*) *ppk2*8-*LexA* driving *LexAop-rCD2::RFP.* (*L*) Merge of *J* and *K*. (*M*) *pain-Gal4* driving *40x UAS-IVS-mCD8::GFP*. (*N*) *Ir94e*-*LexA* driving *LexAop-rCD2::RFP.* (*O*) Merge of *M* and *N*. n = 5-9. (Scale bar, 20 μm.)

*Drosophila pain* is widely expressed in GRNs and displays partial overlap in expression with the gustatory receptors, *Gr47a* and *Gr32a* (19). As a first step to characterize additional gustatory roles for *Drosophila pain*, we explored the specific classes of GRNs that express *pain*. The labellum at the end of the proboscis is decorated with three size classes of sensilla. The large (L-type) and small sensilla (S-type) contain four GRNs, while the intermediate-sized sensilla (I-type) harbor two GRNs. GRNs fall into at least five classes (Table 1; A-E) (14), each of which detects compounds that either promote feeding (+ valence) or inhibit feeding (− valence). We expressed *UAS-mCD8::GFP* under control of the *pain-Gal4* (18), which stained 48.2 ± 3.3 neurons in each of the two labellar palps (Fig. 1*A* and Table 1) and 26.9 ± 2.2 neurons in the first three tarsal segments of the forelegs (*SI Appendix,* Fig. S1*A*).

**Table 1.** *Drosophila pain* coexpression analysis in A-E GRNs

GRN class	Valence	Marker	# neurons labeled by marker	# pain neurons labeled by *marker*	% *pain* neurons labeled by marker	% marker overlapping with *pain*
*pain*	+ or −	*pain-GAL3*	48.2 ± 3.3	48.2 ± 3.3	100.0	100.0
A	+	*Gr64f-LexA*	32.9 ± 0.8	7.7 ± 1.4	16.0	23.4
B	−	*Gr66a-LexA*	21.3 ± 1.5	11.4 ± 1.4	24.0	53.5
C	+	*ppk28-LexA*	15.7 ± 0.3	6.8 ± 0.7	14.1	43.3
D	−	*ppk23-LexA*	20.2 ± 1.8	10.7 ± 1.7	22.2	53.0
E	+	*IR94e-LexA*	9.8 ± 1.1	6.6 ± 0.7	13.7	67.3

Expression of the *pain-Gal4* was examined in bisected labella using the five GRN (A-E) markers. GRNs were counted in serial optical sections (n = 6 to 9) and assessed for colocalization.

We then addressed which GRN classes express the *pain* reporter by performing double-labeling experiments with reporters marking each of the five GRN classes. The highest percentage of *pain*-positive neurons overlapped with B GRNs (Fig. 1 *A*–*C* and Table 1; 23.4%), which respond to chemicals that suppress feeding such as bitter compounds. A similar percentage of *pain*-positive neurons overlapped with the marker for the D GRNs, which respond to salts with a negative valence, and lower percentages with the markers for the A, C, and E GRNs, which respond to attractive compounds, such as sugars, water, and low salt, respectively (Fig. 1 *D*–*O* and Table 1).

### *pain* Is Required for the Fatty Acid Response.

Given its broad expression in GRNs, *pain* has the potential to function beyond its role in detection of AITC (19). To examine additional roles of *pain*, we performed proboscis extension response (PER) assays by transiently applying different chemicals to the labella and then examined the flies’ gustatory reactions. The PER is a behavior in which flies reflexively extend their proboscis when stimulated with an appetitive tastant, signaling willingness to initiate feeding (39). Conversely, aversive compounds suppress the PER when mixed with an attractive stimulus.

**Fig. 2.**
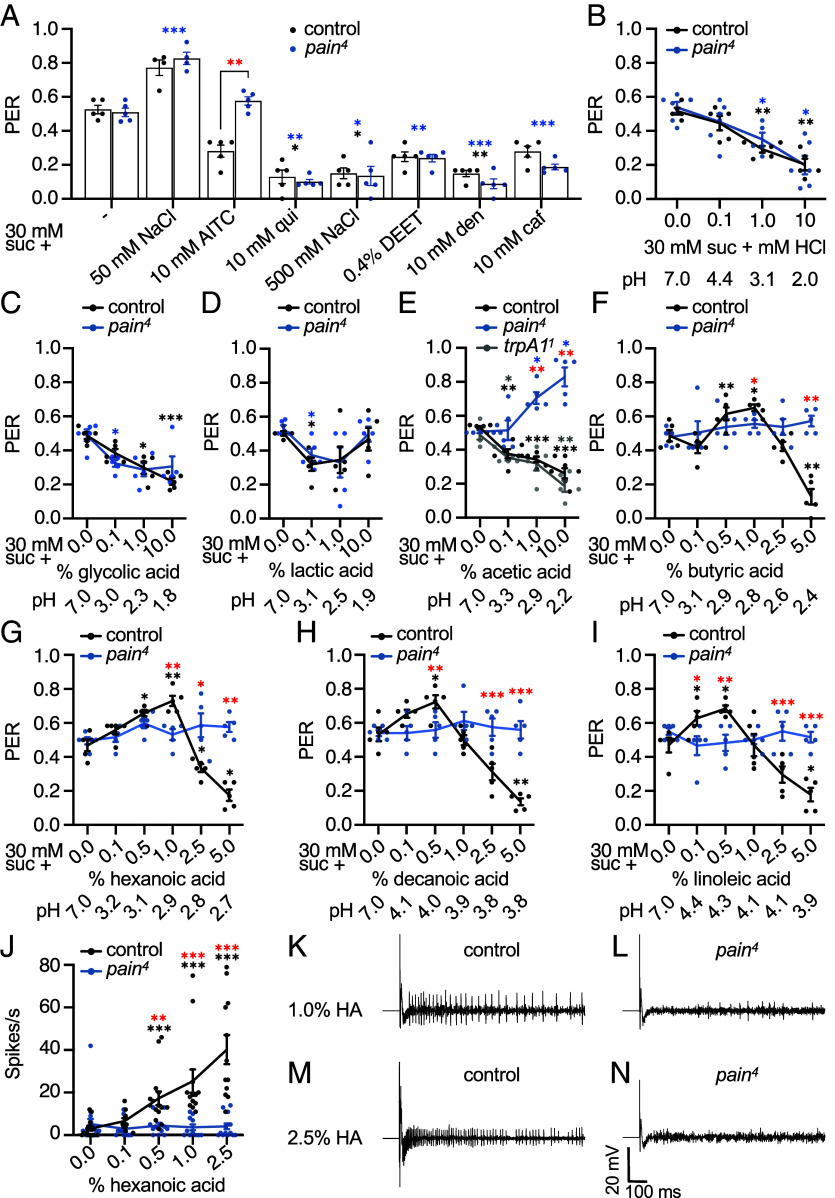
*Drosophila pain* mutants are defective in FA responses. (*A–I*) PER assays performed by stimulating flies with 30 mM sucrose to establish a baseline response, then stimulating with 30 mM sucrose mixed with the indicated compound. Red asterisks indicate significant differences between the control and *pain^4^*. Black asterisks indicate significant differences exhibited by the control flies presented with 30 mM sucrose vs. 30 mM sucrose plus the test compound. Blue asterisks indicate significant differences exhibited by the *pain^4^* flies presented with 30 mM sucrose vs. 30 mM sucrose plus the test compound. Gray asterisks indicate significant differences exhibited by the *trpA1^1^* flies presented with 30 mM sucrose vs. 30 mM sucrose plus acetic acid. (*A*) 30 mM sucrose alone, 50 mM NaCl, 10 mM AITC, 10 mM quinine (qui), 500 mM NaCl, 0.4% DEET, 10 mM denatonium (den), 10 mM caffeine (caf). (*B*) 0.1 to 10 mM HCl. (*C*) 0.1 to 10% glycolic acid. (*D*) 0.1 to 10% lactic acid. (*E*) 0.1 to 10% acetic acid. (*F*) 0.1 to 5% BA. (*G*) 0.1 to 5% HA (C6:0). (*H*) 0.1 to 5% DA (C10:0). (*I*) 0.1 to 5% LA (C18:2). n = 5, N = 10-12 flies/assay. (*J–N*) Extracellular tip recordings performed with HA on S6 sensillum. (*J*) Spike frequencies in response to 0.1 to 2.5% HA. The spikes/second (s) are from 50 to 1,050 ms. The first 50 ms are excluded due to contact artifacts. (*K–N*) Representative tip recording traces. (*K*) Control flies using 1% HA. (*L*) *pain^4^* using 1% HA. (*M*) Control flies using 2.5% HA. (*N*) *pain^4^* using 2.5% HA. n = 10-12. Concentration-dependent changes in mean PERs (panels *A–I*) were analyzed with repeated measures using one-way ANOVA followed by Dunnett’s multiple-comparisons post hoc test. Residuals were checked for normality with the Shapiro–Wilk test and Geisser–Greenhouse corrections were made. These assumptions were violated for the control data in panel *A,* for the *pain^4^* data in panel *F,* and for both control and *pain^4^* data in panel *J*. Therefore, these datasets were evaluated with the repeated measures Friedman test followed by Dunn’s multiple-comparisons post hoc test. In panel *J*, spike frequencies were analyzed using the Kruskal–Wallis test followed by Dunn’s multiple-comparisons post hoc test. For differences between each genotype, Mann–Whitney *U* tests were used. Error bars, SEMs. One asterisk, *P* < 0.05. Two asterisks, *P* < 0.01. Three asterisks, *P* < 0.001.

To attempt to identify additional compounds that depend on Pain for gustatory detection, we focused on *pain^4^*—an allele that we previously generated, which has a deletion removing the region coding for two of the six transmembrane domains and part of the flanking C-terminal region (40). Consistent with previous results, the *pain^4^* mutant elicited the same appetitive response to 30 mM sucrose as control flies (Fig. 2*A*) (19). Addition of low salt (50 mM NaCl) enhanced the PER to the same extent in control and *pain^4^* flies (Fig. 2*A*). In support of previous findings (19), addition of 10 mM AITC to the sucrose reduced the PER in control but not in *pain^4^*, while adding quinine or high NaCl levels to the sucrose produced similar suppression of the PER in both control and *pain^4^* flies (Fig. 2*A*). In addition, there were no differences in suppression of the PER between the control and *pain^4^* flies when we added DEET, denatonium, or caffeine to sucrose (Fig. 2*A*).

We next tested PERs using low pH conferred by either HCl or organic acids. Both control flies and *pain^4^* mutants similarly avoided acidic pHs using various concentrations of HCl (Fig. 2*B*). We assessed carboxylic acids of varying chain lengths and found that control and *pain^4^* flies showed similar PERs to the hydrophilic carboxylic acids, glycolic acid, and lactic acid (Fig. 2 *C* and *D*). Unexpectedly, *pain^4^* exhibited increased PERs toward concentrations of acetic acid (C2:0) that are slightly aversive to control flies (Fig. 2*E*). Given the results with HCl and these carboxylic acids, these latter differences cannot be explained by the low pH levels of the 1% and 10% acetic acid (pHs of 2.9 and 2.2, respectively).

Acetic acid is more hydrophobic than either glycolic acid or lactic acid, and is a short-chain fatty acid (FA). Therefore, we tested whether the *pain^4^* phenotype represented a broader impairment in FA perception. To test this idea, we performed PERs using 30 mM sucrose combined with additional FAs, including another short-chain FA (butyric acid, BA, C4:0), two medium-chain FAs (hexanoic acid, HA, C6:0; and decanoic acid, DA, C10:0), and a long-chain polyunsaturated FA (linoleic acid, LA, C18:2). In control flies, we observed a slight increase in the PER when we added a low level of BA (1%; Fig. 2*F*), and larger increases in the PERs when we added low levels of HA (1%), DA (0.5%), or LA (0.5%) to the sugar (Fig. 2 *G*–*I* and *SI Appendix,* Fig. S2*A*). Addition of higher levels of the FAs (2.5 or 5%) caused reduced PERs (Fig. 2 *F*–*I* and *SI Appendix,* Fig. S2*A*). When we stimulated the tarsi on forelegs with increasing concentrations of HA we saw a dose-dependent increase in PERs to low concentrations (0.5% and 1%) and decreases in PERs to high concentrations (2.5% and 5%; *SI Appendix,* Fig. S2*A*), similar to the responses induced by stimulating labella (Fig. 2*G*). The PERs were not influenced by olfaction since the responses to HA were the same when we surgically removed the two olfactory organs, the antennae and maxillary palps (*SI Appendix,* Fig. S2*B*).

Strikingly, the *pain* mutants were taste-blind to FAs, showing neither increased nor decreased PER across all concentrations tested for BA, HA, DA, and LA via labellar stimulation (Fig. 2 *F*–*I*) and HA via tarsal stimulation (*SI Appendix,* Fig. S2*B*). We focused on HA and tested a second *pain* allele for which the entire *pain* locus is deleted, *pain^pf^* (41). These flies also showed no increased or decreased PERs in response to low or high concentrations of HA, respectively (*SI Appendix,* Fig. S2*C*). This suggests mutation of *pain* causes a generalized deficit in FA detection. Pain belongs to the TRPA subfamily of TRP channels, and a mammalian TRPA channel, TRPA1, is known to detect hydrophobic carboxylic acids (42). However, the *Drosophila trpA1^1^* mutant (43) responded normally to HA (*SI Appendix,* Fig. S2*C*).

### *pain* Is Essential for Fatty Acid-Induced Action Potentials in GRNs.

To test whether the requirement for *pain* for responding to FA reflects a role in GRNs, we assayed for HA-induced action potentials by performing tip recordings, a technique that measures action potentials generated by GRNs housed within individual taste sensilla (44, 45). We inserted an electrode filled with solution containing HA over taste sensilla, thereby allowing both stimulation and recording of neuronal activity. We focused on the S6 sensillum, previously shown to respond to HA in a dose-dependent manner, with low concentrations activating sugar-sensing A GRNs and higher concentrations activating bitter-sensing B GRNs (28, 32). Notably, both A and B GRNs in S6 express *pain* (*SI Appendix,* Fig. S1 *B* and *C*).

We compared HA-induced action potentials using HA levels ranging from 0.1 to 2.5%. Control flies exhibited dose-dependent firing to HA (Fig. 2 *J*, *K*, and *M*). In addition, the amplitude of the spikes was reduced at 2.5% HA vs. 1% HA (Fig. 2 *K* and *M*). Reductions in spike amplitude at higher spike frequencies have been proposed to be due to a phenomenon called depolarization block, which is when a high stimulus can cause persistent depolarization and inactivation of a fraction of voltage-gated Na^+^ channels (46). In contrast to the control, the *pain^4^* mutants exhibited minimal firing at all concentrations (Fig. 2 *J*, *L*, and *N*). However, sucrose, denatonium, low pH, and high salt induced similar action potential frequencies in control and *pain^4^* flies (*SI Appendix,* Fig. S2 *D–O*), consistent with their normal PERs to these stimuli.

To further assess the specificity of *pain* function, we recorded from L4 sensilla, which contain A, C, D, and E GRNs but lack a B GRN. We found that 1% HA activated GRNs in L4 in control flies (*SI Appendix,* Fig. S3*A*), indicating that HA detection is not restricted to B GRNs, consistent with a previous study (32). As with S6 sensilla, *pain^4^* mutants showed a reduction in HA-evoked activity in L4 (*SI Appendix,* Fig. S3 *B* and *C*), demonstrating that *pain* is required for HA activity even in a sensillum that does not house a B GRN. Conversely, *pain^4^* mutants displayed normal responses to 100 mM sucrose and 500 mM NaCl in L4 (*SI Appendix,* Fig. S3 *D*–*I*), further demonstrating that *pain* is not broadly required for detecting tastants.

### Long *pain* Isoform Functions in FA Attraction and Aversion in A and B GRNs.

**Fig. 3.**
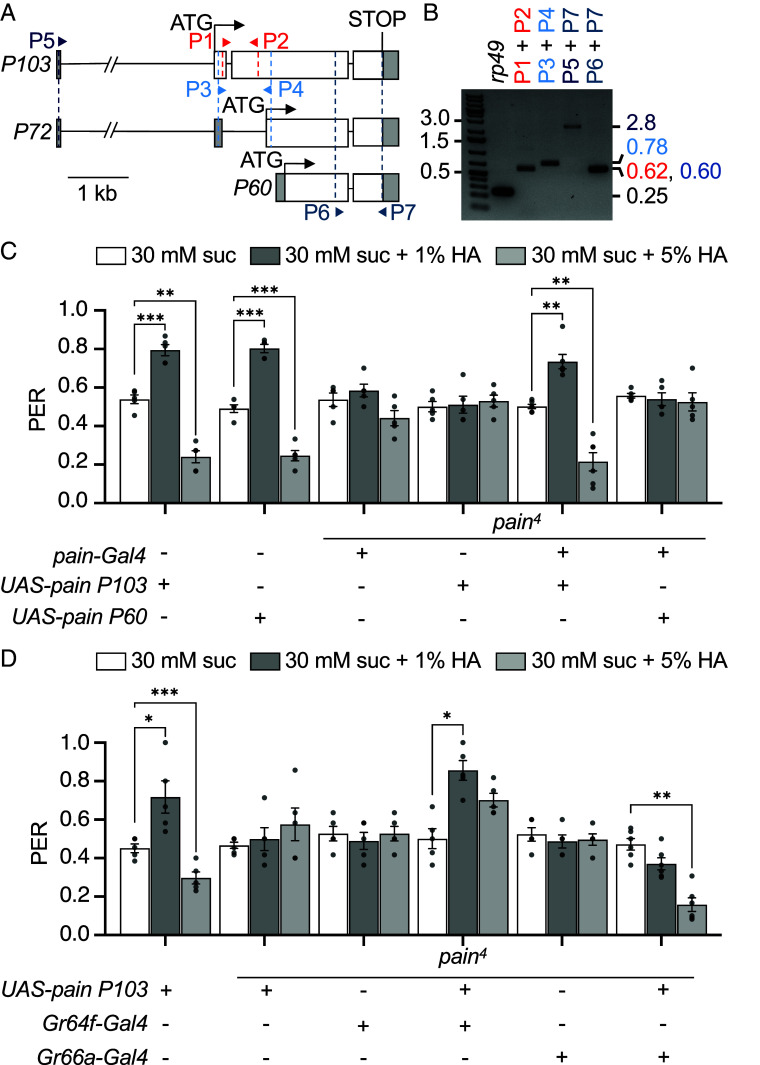
*Drosophila pain P103* is required in A GRNs for attraction to HA and in B GRNs for aversion to HA. (*A*) Schematic depicting the three *pain* mRNA isoforms, *P103*, *P72*, and *P60*. The arrowheads indicate the location of the RT-PCR primers used in *B*. The parallel diagonal slashes indicate 1 kb not shown in the first intron of *P103* and *P72*. (*B*) RT-PCR products prepared from RNA extracted from labella and the indicated primers were fractioned on a 1% agarose gel. The *rp49* control is 248 bp. Band sizes (bp) for the *pain* products using the indicated primer pairs: P1 + P2 (*P103*) = 622 bp, P3 + P4 (*P103*) = 784 bp, P5 + P7(*P103*) = 2.8 kb, P6 + P7 (*P103*, *P72*, and *P60*) = 603 bp. The predicted bands for *P72* were not detected: P3 + P4 = 112 bp, and P5 + P7 = 2.1 kb. DNA size markers (kb) are indicated to the *Left*. (*C* and *D*) PER assays performed by stimulating labella with 30 mM sucrose or 30 mM sucrose with 1% or 5% HA. (*C*) Testing for rescue of the *pain^4^* phenotype by expressing either *UAS-pain P103* or *UAS-pain P60* under the control of the *pain-Gal4*. (*D*) Testing for cell-type rescue of HA attraction and repulsion in the *pain^4^* mutant by expressing *UAS-pain P103* under the control of either the *Gr64f-Gal4* (*A* GRNs) or the *Gr66a-Gal4* (*B* GRNs). n = 5. N = 10-12 flies/n. Concentration-dependent changes in the mean PERs (*C* and *D*) were analyzed with repeated measures using one-way ANOVA followed by Dunnett’s multiple-comparisons post hoc test. Residuals were checked for normality with the Shapiro–Wilk test and Geisser–Greenhouse corrections were made. Error bars, SEMs. *P < 0.05. **P < 0.01. ***P < 0.001.

The *pain* locus is expressed as three mRNA isoforms, encoding three proteins (P103, 913 amino acids; P72, 629 amino acids; P60, 529 amino acids), which differ in the number of N-terminal ankyrin repeats (8, 4, and 2, respectively), but share a common stop codon (47). To address which of the three *pain* mRNA isoforms is required for the attraction and repulsion to low and high levels of HA, we first examined the expression of each mRNA in labella. We designed isoform-specific primers to distinguish between the long mRNA (*P103*; P1 + P2 primers; Fig. 3*A*) and the medium mRNA (*P72*; P3 + P4 primers; Fig. 3*A*). We then prepared cDNAs from RNA isolated from the labella of control flies, and performed RT-PCR. We found that *P103*, encoding the temperature-activated P103 protein isoform (18, 21, 47), was expressed in labella, while *P72* was not (Fig. 3*B*). Due to the overlapping sequences with the other isoforms, we could not determine whether *P60* was expressed.

To distinguish whether *P103* or *P60* functioned in FA taste, we addressed whether a transgene expressing one or the other protein could rescue the *pain* mutant phenotype. We drove expression of either *UAS-pain P103* or *UAS-pain P60* under the control of the *pain-Gal4* in a *pain^4^* background and found that *P103* but not *P60* restored the appetitive PER to 1% HA and suppression of the PER to 5% HA (Fig. 3*C*). These results indicate that the same *pain* isoform functions in the response to low and high concentrations of HA. We then targeted expression of *UAS-pain P103* specifically in A GRNs using the *Gr64f-Gal4*, which restored the appetitive responses to HA in *pain^4^* flies (Fig. 3*D*). We also drove *UAS-pain P103* in B GRNs with the *Gr66a-Gal4*, which rescued the feeding inhibition (Fig. 3*D*). Thus, *P103* mediates responses to both low and high concentrations of HA in A and B GRNs, respectively.

### Expression of *Aedes pain1* in the Major Taste Organs.

Our findings on *Drosophila* Pain raise the possibility that a Pain homolog in *Ae. aegypti* might play a role in detecting FAs. BLAST analysis indicates that there are three mosquito Pain protein homologs—Pain1 (918 amino acids), Pain2 (1,065 amino acids), and Pain3 (1,099 amino acids). The *Aedes pain1* gene (Gene ID: 5568431) encodes Pain1, which shares the highest amino acid identity with *Drosophila* Pain (35%), while Pain2 (ID: 5564686) and Pain3 (ID: 5572129) are 24% and 25% identical, respectively. Based on RT-qPCR (16) and analyses of RNA-seq datasets (48), *pain1* is expressed at the highest levels in *Ae. aegypti* labella/proboscises and tarsi/legs. The *pain2* and *pain3* genes are expressed at 8.8% and 0.8% the levels of *pain1* in the proboscis, and at 10.3% and 1.7% the levels of *pain1* in the legs (48). The higher amino acid homology and expression in gustatory organs relative to the other two *pain* genes suggest that *Aedes pain1* is best among the three candidates for functioning in taste.

To validate the previous findings that *Aedes pain1* is expressed in gustatory organs, we extracted RNA from labella and the first three tarsal segments of the forelegs of *Ae. aegypti* females (*SI Appendix,* Fig. S4*A*) and performed RT-PCR. We amplified a 168 bp product corresponding to *pain1* from labella and tarsi in both males and females (*SI Appendix,* Fig. S4*B*). We also conducted RT-qPCR using *ribosomal protein S7* (*RPS7*) as a reference gene. The results demonstrate that *pain1* is expressed at much higher levels in labella and foreleg tarsi compared to abdomens (*SI Appendix,* Fig. S4*C*).

To spatially localize expression of *pain1* in gustatory organs, we generated a *QF2* gene reporter. Using CRISPR/Cas9, we created *pain1^QF2^* by inserting *T2A-QF2* between residues 254 (Ser) and 255 (Ala), which is N-terminal to the six transmembrane segments (*SI Appendix,* Fig. S4*D*). We then used *pain1*QF2 to drive expression of *QUAS-mCD8:GFP*. To reduce the pigmentation of the dark cuticle and thereby improve detection of the anti-GFP, we introduced these lines in a *yellow* mutant background (49, 50).

**Fig. 4.**
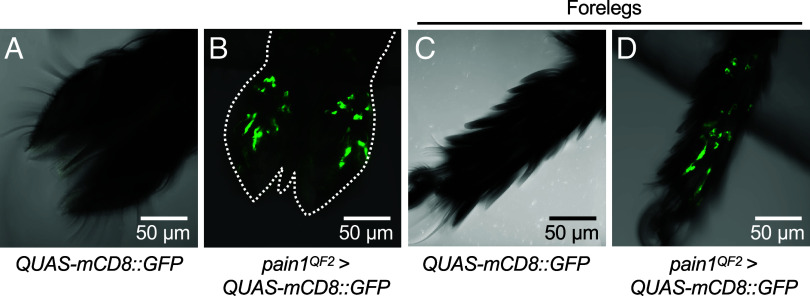
*Aedes pain1^QF2^* reporter expression in the labellum and the most distal foreleg tarsus from females. *pain1^QF2^* was used to drive expression of *QUAS*-m*CD8*::*GFP* in a *yellow* mutant background to reduce the color of the cuticle. GFP was detected by anti-GFP staining (green). The images were acquired using a Zeiss LSM 900 confocal microscope. (*A*) *QUAS*-m*CD8*::*GFP* labellum. (*B*) *pain1^QF2^*>*QUAS*-m*CD8*::*GFP* labellum. (*C*) Distal tarsal segment from a foreleg of a *QUAS*-m*CD8*::*GFP* female. (*D*) Distal tarsus from a foreleg of a *pain1^QF2^*>*QUAS*-m*CD8*::*GFP* female.

We examined both labella and foreleg tarsi for *pain* reporter expression. In each of the two labellar palps there were ≥8 GFP-positive neurons with cell bodies and dendritic processes localized to the anterior region of the proboscis (Fig. 4 *A* and *B*). We also examined leg tarsi since they harbor the first taste organs to contact foods and the surface of hosts, before they initiate a blood meal (10, 51–54). Moreover, mosquito tarsi respond to tastants (10, 54), as is well documented in *Drosophila* (55). We observed ≥12 GFP-positive neurons per first tarsal segment in female forelegs (Fig. 4 *C* and *D*), and ≥11 and ≥10 GFP-positive neurons in the first tarsal segments of the female midlegs and hindlegs, respectively (*SI Appendix,* Fig. S4 *E*–*H*). These results indicate that *pain1* is expressed in a subset of neurons in both the labellum and legs.

### Contribution of Pain1 to Gustatory Deterrence of FAs.

**Fig. 5.**
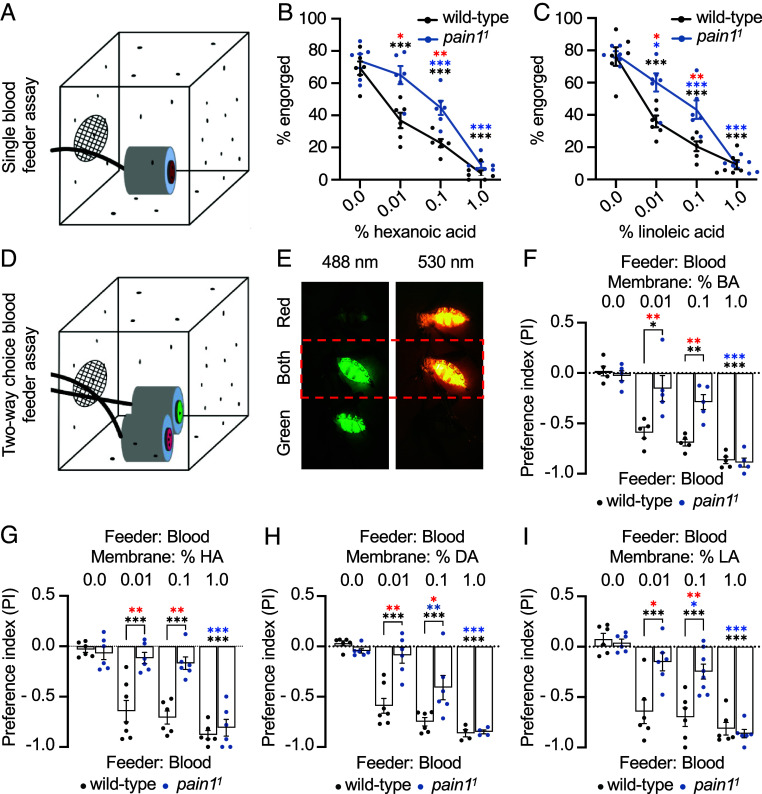
Requirements for *Aedes pain1* for the behavioral response to fatty acids. (*A*) Cartoon of single blood feeder assay setup. (*B* and *C*) Percent engorgement of WT and *pain1^1^* adult females after allowing them to feed for 10 min on a single blood feeder with the indicated percentages of FAs coated on VectaDerm membranes. n = 6. N = 50 mosquitoes/assay. (*B*) Percent HA applied to the membranes. (*C*) Percent LA applied to the membranes. (*D*) Cartoon of two-way choice blood feeder assay. The blood was mixed with either 0.1% rhodamine or 0.1% fluorescein. (*E*) *Ae. aegypti* females that were engorged with blood after two-way choice blood feeder assays. The abdomens were imaged at 488 nm and 530 nm to determine whether the females consumed the blood with either rhodamine (red, *Top* row), or fluorescein (green, *Bottom* row), or if the mosquitoes consumed blood from both feeders (middle row). (*F–I*) Two-way choice blood feeder assays using either WT or *pain1^1^* females. One of the two feeders had blood mixed with either rhodamine or fluorescein and a membrane soaked in water plus the solvent (8% ethanol). The other feeder had blood mixed with the other dye and a membrane coated with the indicated concentration of FAs plus the solvent. We reversed the dyes in different experiments to make sure the dyes had no influence on the outcomes. We then determined the PI. n = 4-7. N = 50 females/assay. (*F*) Tests using blood feeders with one membrane soaked with the indicated percentage of BA and the other membrane soaked with water. (*G*) Tests using blood feeders with one membrane soaked with the indicated percentage of HA and the other membrane with water. (*H*) Tests using blood feeders with one membrane soaked with the indicated percentage of DA and the other membrane soaked with water. (*I*) Tests using blood feeders with one membrane soaked with the indicated percentage of LA and the other membrane soaked with water. The black asterisks indicate significant differences exhibited by WT mosquitoes tested with 0% vs. 0.01%, 0.1%, and 1.0% FAs. The blue asterisks indicate significant differences exhibited by *pain1^1^* tested with 0% vs. 0.01%, 0.1%, and 1.0% FAs. The red asterisks indicate significant differences between WT and *pain1^1^*. The statistics between WT and *pain1* mutant were performed with Mann–Whitney tests. The statistics within each genotype for different concentrations of FAs were checked with one-way ANOVA followed by Dunnett’s multiple-comparisons post hoc test. Residuals were tested for normality using the Shapiro–Wilk test and for equal variances with the Brown–Forsythe test (panels *B*, *C*, and *F–I*). For differences between each genotype, Mann–Whitney *U* tests were used. Error bars, SEMs. One asterisk, *P* < 0.05. Two asterisks, *P* < 0.01. Three asterisks, *P* < 0.001.

Given that *Drosophila pain* mediates taste aversion to high FA concentrations, we hypothesized that *Aedes pain1* may play a similar role. To test this, we used a single blood feeder assay containing warm sheep blood. We lined the feeder with an artificial membrane (VectaDerm) that mimics many more of the features of human skin (porosity, pH, and elasticity) than commercial Hemotek membranes. VectaDerm also permits coating the membranes with FAs by soaking them in solutions with different concentrations of FAs. When we exposed 50 mated wild-type (WT) females for 10 min to a 37 °C blood feeder (Fig. 5*A*) with no HAs on the membranes, 69.1 ± 3.9% engorged on blood (Fig. 5*B*). We coated the membranes with several concentrations of HA (0.01%, 0.1%, and 1%) and LA (0.01%, 0.1%, and 1%) and found that WT females showed a dose-dependent decrease in feeding in the presence of FAs (Fig. 5 *B* and *C*).

The observation that fewer females fed on FA-soaked membranes, raised the question as to whether females land on these feeders to the same extent as water-soaked membranes, but refrain from blood feeding if they sense LA. To address this question, we soaked membranes in 1% LA plus the solvent (8% ethanol) and 0.1% rhodamine B to mark tissues that make contact with the membrane. As a control, we soaked the membranes in water plus the solvent and 0.1% rhodamine B. We then performed single feeder assays. ~87% of the females landed on the feeder with a membrane soaked in water (*SI Appendix,* Fig. S5*A*), as evidenced by fluorescent dye on tarsi and the tip of labella (*SI Appendix,* Fig. S5*B*). Among these landed females, ~76% engorged on blood (*SI Appendix,* Fig. S5*A*). ~85% females landed on blood feeders coated with 1% LA but only ~2% engorged (*SI Appendix,* Fig. S5*A*). Thus, females land on membranes with LA but choose not to feed. Moreover, as soon as they touch the LA-containing membrane they fly away (Movie S1), indicating that stimulation of pharyngeal GRNs is not required for this aversive behavior.

To further assess WT feeding preferences when presented with FAs, we subjected 50 female mosquitoes to two-way choice blood feeder assays. This assay provides mosquitoes with the opportunity to choose between two blood feeders—one with a VectaDerm membrane coated with FA and the other membrane soaked in the same solution without any FA (Fig. 5*D*). To distinguish which option the mosquitoes selected, we spiked the blood with easily distinguishable fluorescent dyes: rhodamine (red) or fluorescein (green) (Fig. 5*E*). These dyes were balanced such that they did not influence mosquito preferences on their own, as evident by the preference index (PI) of ~0 when both membranes were soaked in the same FA-free solvent (Fig. 5*F*). WT females avoided 0.01, 0.1, and 1% BA (Fig. 5*F*). Similarly, they overwhelmingly preferred the HA-free feeder, even when one option was coated with only 0.01% HA (Fig. 5*G*).

The preceding data indicate that HA is highly aversive. However, FAs are present on human skin. Therefore, the feeding deterrence caused by FAs might be eliminated in the presence of multiple host cues. The blood feeder is warmed to 37 °C and therefore provides host cues in the form of thermal infrared, convection, and conduction heat. To add additional host cues, we conducted two-way choice blood feeding assays in which both blood feeders were presented in the presence of CO_2_ or CO_2_ plus human odor; however, only one blood feeder membrane was soaked in HA. Even under these conditions, WT mosquitoes maintained aversion to 0.01%, 0.1%, and 1% HA (*SI Appendix,* Fig. S5*C*), indicating that FA aversion is not overridden by multiple host-derived cues.

To test whether *pain1* is necessary for FA repulsion, we generated two mutant alleles. The first is the *pain1^QF^* described above that interrupts the *pain1* gene with an insertion of the *QF2* reporter and a *DsRed* transgenic marker (*SI Appendix*, Fig. S4*D*). The second *pain1^1^* allele disrupts the *pain1* gene with introduction of *DsRed* in the region encoding residue 454 (Tyr; *SI Appendix*, S4*D*). We outcrossed both the *pain1^QF2^* and the *pain1^1^* lines to a WT (LVP) strain for ≥5 generations and created homozygous mutant lines. Because the insertions interrupted the coding regions N-terminal to the six transmembrane domains in Pain, they are likely to be null mutations. Furthermore, the insertions reduced the *pain1* RNAs below the level of detection by RT-PCR (*SI Appendix*, S4 *I*–*K*), presumably due to nonsense-mediated mRNA decay (56). Mutations in *Drosophila pain* disrupt gustatory aversion to AITC. Therefore, we first checked whether *Aedes pain1* also functions in AITC repulsion. Using two-way choice blood feeder assays, control mosquitoes strongly avoided consuming 10 mM AITC, while this effect was significantly reduced in the *pain1^1^* mutants (*SI Appendix,* Fig. S5*D*).

To characterize the effects of loss of *pain1* on the gustatory avoidance of FAs, we performed single blood feeder and two-way choice blood feeder assays. Using the single-blood feeder assay, *pain1^1^* displayed significantly reduced aversion to 0.01% and 0.1% HA relative to WT females (Fig. 5*B*). The percent of *pain1^1^* females that engorged with 0.01% on the membrane was not significantly different from *pain1^1^* presented with an HA-free membrane.

The effect of the *pain1^1^* mutation was more pronounced when the mosquitoes had a choice between two feeders, one with and the other without HA. In this case, *pain1^1^* exhibited indifference between the blood feeder with no HA vs. the blood feeders with membranes laced with either 0.01% or 0.1% HA (Fig. 5*G*). However, *pain1^1^* avoided 1% HA as robustly as WT in either the single blood feeder assay (Fig. 5*B*) or the two-way choice assay (Fig. 5*G*). Using the two-way choice assay, the *pain1^QF2^* mutant also displayed significantly diminished aversion to 0.1% HA (*SI Appendix,* Fig. S5*E*), similar to *pain1^1^* females (Fig. 5*G*). We checked another medium-chain FA, DA (C10:0). WT mosquitoes strongly avoided DA, and this repulsion was reduced by the *pain1^1^* mutation (Fig. 5*H*). These data demonstrate that *Aedes pain1* is required for avoiding both of the medium-chain FAs during blood feeding, except at the highest concentrations tested.

In *Drosophila*, FA taste discrimination is partially dependent on chain length (57). Therefore, we addressed whether *Aedes* Pain1 impacts the response to LA (C18:2), which is a long chain FA. In assays with a single blood feeder, WT females avoided LA in a dose-dependent manner whereas the *pain1^1^* mutants exhibited a reduced repulsion to 0.01% and 0.1% LA but not 1%, (Fig. 5*C*). The *pain1^1^* mutant also showed a significant reduction in LA avoidance using the two-way choice assay (Fig. 5*I*). The *pain1^QF^* flies exhibited a reduced avoidance to LA similar to *pain1^1^* (*SI Appendix,* Fig. S5*F*). The *pain1^1^* mutant was also defective in the aversion to BA (C4:0), which is a short-chain FA (Fig. 5*F*). These results indicate that *Aedes pain1* plays a role in detecting FAs across chain lengths.

To determine whether a role for Pain1 in FA detection extends to nectar feeding, we performed two-way choice assays. We offered females 20 mM sucrose alone or sucrose mixed with different concentrations of LA in alternating positions in half the wells in a 96-well microtiter dish (*SI Appendix,* Fig. S5*G*). One food contained sulforhodamine B and the other brilliant blue FCF. To determine which food the mosquitoes consumed, we inspected their abdomens for red, blue, and purple colors (if both options are consumed; *SI Appendix,* Fig. S5*H*). WT females avoided the LA-containing sucrose, while *pain1^1^* mutants showed greatly diminished aversion to sucrose laced with either 0.01% or 0.1% LA (*SI Appendix,* Fig. S5*I*). Collectively, these results demonstrate that *Aedes pain1* is required for detecting FAs during both blood and nectar feeding, and that it plays a critical role in aversive gustatory responses.

### *pain1* Mutants Display Defects in FA Aversion Independent of Olfactory Detection.

Mosquitoes prefer some humans over others and this is due in part to the olfactory attraction of elevated levels of certain FAs on skin in the context of other host chemicals (22). However, the precise levels of these FAs and other relevant surface compounds that render the FAs attractive have not been described. On the other hand, high concentrations of some FAs can induce aversive olfactory responses by mosquitoes (23, 24).

Given the role of *pain1* in mediating aversive taste responses to FAs, as well as expression of *pain1* mRNA in antennae (48), we examined whether *pain1* also contributes to olfactory responses by FAs. We employed a Y-tube olfactometer to assess odor-driven preference, using a design similar to that described previously (58) (*SI Appendix,* Fig. S6*A*). Each arm of the olfactometer contained an odor source and a trap for capturing mosquitoes following their decision to turn left or right. To test olfactory responses to LA, we presented female mosquitoes with two odor streams: one contained 5% CO_2_ alone, which activates the females (59), and the other with CO_2_ combined with different concentrations of HA or LA. WT mosquitoes exhibited significant olfactory attraction to levels of HA or LA (0.1% and 1%) that result in gustatory repulsion (*SI Appendix,* Fig. S6 *B* and *C*), consistent with roles of FAs as attractive odor cues (5, 22, 60). Notably, *pain1^1^* mutants displayed attraction to the odor of either HA or LA comparable to WT (*SI Appendix,* Fig. S6 *B* and *C*). These results indicate that *pain1* is not required for olfactory detection of these levels of FAs and that the *pain1*-dependent gustatory aversion is not due to olfactory repulsion.

To further investigate a potential role of the olfactory system, in mediating the gustatory repulsion that depends on *pain1*, we performed field recordings (electroantennograms, EAGs; *SI Appendix,* Fig. S6*D*). We exposed female antennae to paraffin oil (solvent control) and 1% HA and found that the EAG responses were comparable between WT and *pain1^1^* (*SI Appendix,* Fig. S6 *E*–*G*).The behavioral and electrophysiological data indicate that *pain1* is not required for olfactory detection of FAs levels that cause gustatory repulsion.

### Labellar GRNs Depend on Pain1 to Respond to FAs.

**Fig. 6.**
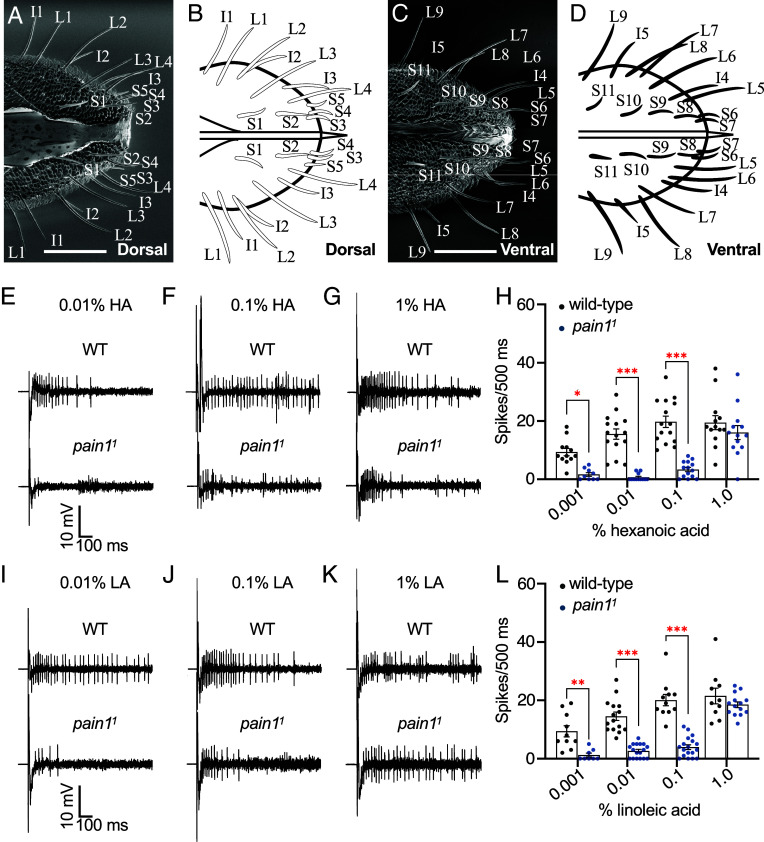
FA-induced action potentials recorded from sensilla on labella from female *Ae. aegypti*. (*A*) SEM image of a female labellum (dorsal view). The dorsal side of the labellum reveals four long-type sensilla (L1-L4), three intermediate-type sensilla (I1-I3), and five small-type sensilla (S1-S5). (*B*) Cartoon of the dorsal view of the labellum shown in *A.* (*C*) SEM image of a female labellum (ventral view). This side of the labellum shows five long-type sensilla (L5-L9), two intermediate-type sensilla (I4-I5), and six small-type sensilla (S6-S11). (*D*) Cartoon of the ventral view of the labellum shown in *C*. (Scale bar, 50 μm.) (*E–L*) Tastant-induced action potentials were assayed using tip recordings on L3 sensilla from WT and *pain1^1^* female labella. (*E*) Representative traces in response to 0.01% HA. (*F*) Representative traces in response to 0.1% HA. (*G*) Representative traces in response to 1% HA. (*H*) Average spikes/500 ms with the indicated concentrations of HA. Due to adaptation, we present the frequencies for 500 ms rather than 1 s. n = 9-15. (*I*) Representative traces in response to 0.01% LA. (*J*) Representative traces in response to 0.1% LA. (*K*) Representative traces in response to 1% LA. (*L*) Average spikes/500 ms with the indicated concentrations of LA. n = 8-15. The red asterisks indicate significant differences between WT and *pain1^1^*. For differences between each genotype, Mann–Whitney *U* tests were used. Error bars, SEMs. **P < 0.01. ***P < 0.001.

Gustatory sensilla distributed on the labella of *D. melanogaster* (61, 62) and *Aedes albopictus* (9) are classified into three sizes: long (L), intermediate (I), and small (S). The gustatory sensilla on the labella of *Ae. aegypti* have also been examined previously, with a focus on the long sensilla (63). To establish an anatomical map of the complete set of labellar taste sensilla, we performed scanning electron microscopy (SEM). We focused on females since it has been reported that the position and numbers of taste sensilla are consistent between males and females (63). We imaged the dorsal half of 36 labella and the ventral half of 27 labella (Fig. 6 *A*–*D*). We found that 25 gustatory sensilla decorated each palp, and that they fell into L, S, and I types consistent with *Ae. albopictus* and *D. melanogaster*. The dorsal side of each labellum includes four L-type (L1–L4), three I-type (I1–I3), and five S-type (S1-S5) sensilla (*SI Appendix,* Fig. S7*A*), and the ventral side has five L-type (L5-L9), two I-type (I4 and I5), and six S-type (S6-S11; *SI Appendix,* Fig. S7*A*).

To determine whether *pain1* is required for FA-induced action potentials, we performed tip recordings on females. We screened eight sensilla (L1–L4, I2, I3, S4, S5) that were more easily accessible for recording based on their location near the margin. We found that L3 and L4 sensilla produced the highest frequencies of action potentials in response to 0.1% LA (*SI Appendix,* Fig. S7*B*). Using the L3 sensillum from the dorsal orientation, we tested four HA concentrations (0.001%, 0.01%, 0.1%, and 1%) and found that WT mosquitoes exhibited dose-dependent increases in spike frequency (Fig. 6 *E*–*H*).

In contrast to WT, *pain1^1^* mutants showed significantly reduced FA responses. The action potential frequencies were greatly reduced at 0.001%, 0.01%, and 0.1% HA, but retained strong responses at 1% (Fig. 6 *E*–*H*). We performed recordings from L3 sensilla from *pain1^QF2^*, which also exhibited significantly reduced neuronal responses to 0.01% and 0.1% HA (*SI Appendix,* Fig. S7*C*). We obtained similar results with LA. WT showed dose-dependent responses to 0.001%, 0.01%, 0.1%, and 1% LA, whereas the *pain1^1^* mutant displayed significantly reduced responses at 0.001%, 0.01%, and 0.1%, but normal responses at 1% (Fig. 6 *I*–*L*). We confirmed this phenotype with *pain1^QF2^*, which also showed significantly reduced neuronal responses to 0.01% and 0.1% LA (*SI Appendix,* Fig. S7*D*). These data demonstrate that *pain1* is required for detecting lower concentrations but not the highest concentrations of FAs. In contrast to effects of the *pain1* mutations on FA-induced action potentials, control and *pain1^1^* mosquitoes displayed similar frequencies of action potentials upon application of 100 mM sucrose or 10 mM caffeine in the recording pipet (*SI Appendix,* Fig. S7 *E* and *F*). To address the question as to whether the GRNs adapt to stimulation with sucrose or HA we performed tip recordings with three consecutive stimulations (at 3 s intervals) with either 100 mM sucrose or one of three concentrations of HA (0.01%, 0.1%, and 1%). WT females elicited significantly lower spike frequencies in response to the second and third sucrose stimulations compared to the first (*SI Appendix,* Fig. S7*G*). In the case of 1% HA, there were significant decreases in spikes in response to the second and third stimulations; however, at the lower concentrations the decreases were significant only during the third (0.01% HA) or second stimulations (0.1% HA; *SI Appendix,* Fig. S7*G*).

While Pain1 is required for FA-induced action potentials, it does not appear to be sufficient for responding directly to FAs, since when we expressed either *Drosophila* Pain or *Aedes* Pain1 in HEK293 cells and performed Ca^2+^ photometry, they were not activated by 100 μM LA, but were thermally activated by a pulse at 42 °C (*SI Appendix,* Fig. S8). These results are reminiscent of the previous observations on *Drosophila* Pain, which is required for the gustatory avoidance of AITC (19), but is not directly activated by AITC (21). Consistent with these findings, we did not observe any impact of 1 mM AITC in HEK293 cells expressing either Pain or Pain1 (*SI Appendix,* Fig. S8).

## Discussion

This work identifies Pain homologs in *Drosophila* and *Aedes* as evolutionarily conserved TRP channels required for the behavioral responses to contact FAs. In support of these conclusions, mutations in either *Drosophila pain* or *Aedes pain1* significantly reduce the gustatory reactions to FAs. Moreover, both of these TRP channels are necessary for FA-induced action potentials. The gustatory aversion to FAs documented here does not appear to be influenced by olfactory detection of FAs. The gustatory repulsion to FAs was the same in intact *Drosophila*, and in fruit flies in which the olfactory organs, the antennae and maxillary palps, were removed. Furthermore, in *Ae. aegypti*, the olfactory behavior and electrophysiological responses to levels of FAs that cause gustatory repulsion were indistinguishable between control mosquitoes and *pain1* mutants.

The demonstration that Pain1 is required for the gustatory repulsion to FAs represents the first signaling protein required for aversive taste in a mosquito. Moreover, Pain homologs are not broadly tuned to tastants because, with the exception of AITC, the responses to other compounds tested were not affected in the *pain* and *pain1* mutants. The findings that Pain and Pain1 both function in the gustatory repulsion of FAs despite the ~240 My since *Drosophila* and *Aedes* had a common ancestor (http://www.timetree.org/) underscores the importance of avoiding consumption of FAs in these dipterans. This is particularly notable in *Aedes*, in which even the lowest concentrations of FAs tested induced gustatory avoidance.

While *Aedes* Pain1 is required for the gustatory avoidance of FAs, there are likely to be other mosquito receptors/channels that function in GRNs for this avoidance. FAs are insufficient to activate *Aedes* Pain1 *in vitro*, which is also the case with *Drosophila* Pain. TRPs are tetrameric channels and it is possible that another TRP channel forms a heteromultimer with Pain1, thereby conferring direct activation by FAs. Alternatively, there are many examples of signaling cascades that are initiated by G-protein-coupled receptors (GPCRs) that culminate with activation of TRP channels, especially GPCRs that couple to Gq and phospholipase C. Therefore, it is also plausible that a GPCR that couples to Gq may be the FA receptor that indirectly leads to activation of Pain and Pain1. Consistent with latter possibility there are multiple mammalian GPCRs that are activated by short, medium, or long-chain FAs (FFAR1-4), some of which initiate Gq/PLC signaling pathways (64). Moreover, the PLC encoded by *norpA* functions in FA attraction in *Drosophila* (27, 28). However, a role for a PLC in FA avoidance has not been described. GRs participate in FA aversion (32, 33), and therefore might be considered candidate receptors that signal in concert with Pain channels. However, evidence that GRs are direct receptors for FAs is lacking, and it is not clear how activation of GRs might couple to gating of a TRP channel. Nevertheless, since *Drosophila pain* is required for attraction to low levels of FA and repulsion to high FA levels through expression of different classes of GRNs (A and B GRNs, respectively), distinct receptors may be the primary FA detectors in A and B GRNs. Along these lines, it is notable that different GRs and IRs are involved in attraction and avoidance of FAs through expression in either A GRNs (27–30) or B GRNs (32, 33), respectively.

A question arises as to why contact with FAs, such as HA, are aversive. Several studies show that FAs, including HA, can be toxic to a variety of fly species (31–33). FAs are hydrophobic, and due to the highly hydrophobic properties of the surface of their legs, mosquitoes, such as *Ae. aegypti*, find hydrophobic surfaces repellent, thereby reducing contact time (65). Thus, while FAs can be highly attractive olfactory cues that help guide mosquitoes hosts, contact with FAs by the taste system inhibits feeding.

Finally, it is notable that unlike many TRP channels that have homologs in both vertebrates and invertebrates, there are no Pain channels in vertebrates. Rather, Pain homologs are expressed in many insect species and a subset of other arthropods such as crustaceans (35–38). In view of the presence of Pain in mosquito-disease vectors but not mammals, and that it participates in gustatory aversion, Pain represents an intriguing target for the development of a class of insect repellents to suppress biting and the spread of insect-borne disease.

## Materials and Methods

### *Drosophila* Husbandry and Stocks.

Fruit flies were reared in vials or bottles containing cornmeal-yeast media at 25 °C in a 65% humidified chamber under 12 h light/12 h dark cycles. All mutant lines were outcrossed with control flies (*w^1118^*) for ≥5 generations. Details of the fly stocks are provided in *SI Appendix*.

### Mosquito Stocks, Husbandry, and Creation of *pain1* Mutant Alleles.

The Liverpool (LVP) strain of *Ae. aegypti* was the WT control. All mutant mosquito lines were outcrossed to the LVP strain for ≥5 generations. Mosquitoes were reared at 28 °C and 80% relative humidity under 14:10 h light:dark cycles. The *pain1^QF2^* and *pain1^1^* alleles were generated using CRISPR-mediated homology-directed repair. Details of the mosquitoes propagation and egg collection, and creation of the *pain1* alleles are provided in *SI Appendix*.

### cDNA Synthesis and RT-qPCR.

cDNAs for RT-qPCR were prepared from RNA isolated from proboscises from fruit flies, and from proboscises, forelegs, abdomens from female mosquitoes. Details of the synthesis and RT-qPCR are provided in *SI Appendix*.

### PER Assays.

5- to 7-d-old *Drosophila* were starved for 18 to 22 h on water-soaked Kimwipes. Prior to performing the labellar and tarsal PER assays, the flies were satiated with water by touching their labella or tarsi with a Kimwipe wick soaked in water. Proboscis extension was scored as 1, and no response was scored as 0. Flies were tested with 30 mM sucrose with the appropriate solvent for the assay, and then 30 mM sucrose plus the taste compound of interest. Details of the PER assays and the chemicals used for the assays are provided in *SI Appendix*.

### Blood Feeding, Landing Assays on Blood Feeders, and Nectar-Feeding Assays.

Each blood feeding assay (10 min) was with 50 mated, female mosquitoes. To perform the assays, VectaDerm was soaked in water or water containing FAs and applied to feeders with sheep blood. For the two-way choice assays, one feeder also had 0.1% fluorescein dye and the other had 0.1% rhodamine B dye. To identify mosquitoes that landed on a blood feeder, we examined them for the presence of dye on their legs and labellum. Nectar feeding assays were performed for 3 h in 96-well plates with two food options—one with 20 mM sucrose solution plus either sulforhodamine or brilliant blue FCF dye, and the other with 20 mM sucrose mixed with the indicated concentration of FAs and the other dye. Assay details are provided in *SI Appendix*.

### Olfactory Assays Using a Y-Tube Olfactometer.

Y-tube olfactometer assays were conducted at 28 °C and 80% relative humidity. 20 mated, 7 to 10-d-old female mosquitoes were introduced into the holding chamber and acclimated for 10 min before released. Cups with the stimulus and control solutions were placed inside the sample-holding chambers. To initiate the assays, a 5% CO_2_ source was turned on, the mosquitoes were allowed to enter the olfactometer and choose between the two arms for 10 min. The numbers of mosquitoes present in the test and control arms were counted. Details of the construction of the Y-tube olfactometer, execution of the assay, and calculation of the preference indexes are provided in *SI Appendix*.

### Immunostaining and SEM.

Labella and tarsi from 5 to 7-d-old flies 5 to 10-d-old female mosquitoes were fixed in 4% paraformaldehyde, washed, blocked, and incubated with primary antibodies overnight at 4 °C. The samples were washed, incubated with secondary antibodies overnight at 4 °C, mounted using Vectashield, and imaged using a Zeiss LSM 900 confocal microscope. Images were processed using Zen Blue software and ImageJ. SEMs of mosquito labella were captured with a Thermo Fisher Scientific ApreoS 13.5.0. Details of the immunostainings, SEMs, and the source of reagents are provided in *SI Appendix*.

### Tip Recordings and EAG Recordings.

Tip recordings were performed on 5- to 10-d-old fruit flies or female mosquitoes. The recording electrodes were inserted over the sensilla and signals were amplified and digitalized with an IDAC-4 data acquisition device and Autospike software (Syntech Oeckenfels GmbH). The electrical signals were amplified using a Syntech signal connection interface box. The spikes were analyzed with Autospike 3.1 (Syntech).

EAG recordings were performed on female mosquitoes (aged 6 to 8 d). The distal tips of both antennae were trimmed and the remaining antennae were dipped in electrode gel to adhere them together. Excised heads were positioned on a reference electrode filled with saline solution. Both antennae were inserted into a recording electrode containing the saline solution. Odorant was applied to a filter paper placed inside a Pasteur pipet, which was then introduced into a continuous air stream. The responses were amplified and recorded using an IDAC-4 data acquisition system with EAGpro software (Syntech Oeckenfels GmbH). Details of procedures and reagents are provided in *SI Appendix*.

### Cell Culture and Ca^2+^ Photometry.

HEK293 cells were maintained in high-glucose DMEM (Gibco) supplemented with 10% FBS and seeded onto poly-L-lysine-coated coverslips 24 h before transfection. The *Drosophila pain* plasmid (pCMV-*dPain*) was previously described (21). *Ae. aegypti pain1* was synthesized and inserted into the pcDNA3.1. HEK293 cells were transfected with either pCMV-*dPain* or pcDNA3.1-*Aapain1*. To avoid thermal activation of the channels, cells were transferred to 33 °C for 36 h prior to imaging as described (21). 36 to 40 h posttransfection, HEK293 cells were incubated with Fluo-8 AM dye, stimulated with LA, AITC and 42 °C and fluorescence was excited at 488 nm and collected at 510 nm. Details are provided in *SI Appendix*.

### Quantification and Statistical Analysis.

All error bars represent SEMs. The Mann–Whitney *U* test was applied for two-group comparisons (i.e., the difference between controls and mutants). Differences among multiple groups (i.e., for testing concentration dependence in blood feeding assays within genotypes where separate groups are compared to the same control) were analyzed by one-way ANOVA followed by Dunnett’s post-hoc test. Residuals were tested for normality using the Shapiro–Wilk test and for equal variances with the Brown–Forsythe test. For datasets that violated parametric assumptions, the Kruskal–Wallis test with Dunn’s multiple-comparison adjustment was performed for multi-group analyses. Paired datasets generated with the PER assays were analyzed using repeated-measures one-way ANOVA with the Geisser–Greenhouse correction. Residuals were checked for normality using the Shapiro–Wilk test and sphericity (equal variability) was not assumed. If the Geisser–Greenhouse ε < 0.75, the corrected *P*-values were used. We used paired Student’s *t* tests to compare the frequencies of action potentials between the first vs. either the second or third stimulations with the same chemical. Analyses were performed using GraphPad Prism 10. One asterisk, *P* < 0.05. Two asterisks, *P* < 0.01. Three asterisks, *P* < 0.001. Additional details are provided in *SI Appendix*.

## Supplementary Material

Appendix 01 (PDF)

Movie S1. Slow-motion landing of *Ae. aegypti* females on a blood feeder with 1% linoleic acid (LA). Video footage was recorded at 60 fps using a Canon EOS Rebel T6X using an Altura super macro lens and played back via Clipchamp at 6 fps. The video shows females approaching, landing, and making contact with their tarsi and labellum on the feeders. The video illustrates reduced sustained feeding if LA is present, despite initial landing, consistent with LA-induced gustatory avoidance on contact.

## Data Availability

Source and raw data have been deposited in Dryad (DOI: https://doi.org/10.5061/dryad.qrfj6q5ww) (66). All other data are included in the manuscript and/or supporting information.

## References

[1] C. Montell, The sensory arsenal mosquitoes use to find us. Trends Parasitol. **41**, 591–602 (2025). 40447468 10.1016/j.pt.2025.05.004 PMC12352024

[2] C. J. McMeniman, R. A. Corfas, B. J. Matthews, S. A. Ritchie, L. B. Vosshall, Multimodal integration of carbon dioxide and other sensory cues drives mosquito attraction to humans. Cell **156**, 1060–1071 (2014). 24581501 10.1016/j.cell.2013.12.044 PMC4007582

[3] F. van Breugel, J. Riffell, A. Fairhall, M. H. Dickinson, Mosquitoes use vision to associate odor plumes with thermal targets. Curr. Biol. **25**, 2123–2129 (2015). 26190071 10.1016/j.cub.2015.06.046 PMC4546539

[4] M. Carnaghi, S. R. Belmain, R. J. Hopkins, F. M. Hawkes, Multimodal synergisms in host stimuli drive landing response in malaria mosquitoes. Sci. Rep. **11**, 7379 (2021). 33795798 10.1038/s41598-021-86772-4 PMC8016827

[5] I. V. Coutinho-Abreu, J. A. Riffell, O. S. Akbari, Human attractive cues and mosquito host-seeking behavior. Trends Parasitol. **38**, 246–264 (2022). 34674963 10.1016/j.pt.2021.09.012 PMC10789295

[6] A. Chandel , Thermal infrared directs host-seeking behavior in *Aedes aegypti* mosquitoes. Nature **633**, 615–623 (2024). 39169183 10.1038/s41586-024-07848-5 PMC11410652

[7] W. J. Laursen, R. Tang, P. A. Garrity, Hunting with heat: Thermosensory-driven foraging in mosquitoes, snakes and beetles. J. Exp. Biol. **226**, jeb229658 (2023). 37382467 10.1242/jeb.229658 PMC10323236

[8] W. J. Laursen , Humidity sensors that alert mosquitoes to nearby hosts and egg-laying sites. Neuron **111**, 874–887 (2023). 36640768 10.1016/j.neuron.2022.12.025 PMC10023463

[9] L. S. Baik , Mosquito taste responses to human and floral cues guide biting and feeding. Nature **685**, 639–646 (2024). 10.1038/s41586-024-08047-y PMC11578787 39415007

[10] L. S. Baik, J. R. Carlson, The mosquito taste system and disease control. Proc. Natl. Acad. Sci. U.S.A. **117**, 32848–32856 (2020). 33372129 10.1073/pnas.2013076117 PMC7776869

[11] S. Xiao, L. S. Baik, X. Shang, J. R. Carlson, Meeting a threat of the Anthropocene: Taste avoidance of metal ions by *Drosophila*. Proc. Natl. Acad. Sci. U.S.A. **119**, e2204238119 (2022). 35700364 10.1073/pnas.2204238119 PMC9231609

[12] S. Y. Zhao , Gustatory receptor 11 is involved in detecting the oviposition water of Asian tiger mosquito, *Aedes albopictus*. Parasit. Vectors **17**, 367 (2024). 39210465 10.1186/s13071-024-06452-w PMC11363565

[13] B. J. Matthews, M. A. Younger, L. B. Vosshall, The ion channel *ppk301* controls freshwater egg-laying in the mosquito *Aedes aegypti*. eLife **8**, e43963 (2019). 31112133 10.7554/eLife.43963 PMC6597239

[14] C. Montell, *Drosophila* sensory receptors—A set of molecular Swiss army knives. Genetics **217**, 1–34 (2021). 10.1093/genetics/iyaa011 PMC8045702 33683373

[15] Y. D. Chen, A. Dahanukar, Recent advances in the genetic basis of taste detection in *Drosophila*. Cell. Mol. Life Sci. **77**, 1087–1101 (2020). 31598735 10.1007/s00018-019-03320-0 PMC7125039

[16] J. T. Sparks, J. D. Bohbot, J. C. Dickens, The genetics of chemoreception in the labella and tarsi of *Aedes aegypti*. Front. Neural Circuits **48**, 8–16 (2014). 10.1016/j.ibmb.2014.02.004 24582661

[17] K. Venkatachalam , Motor deficit in a *Drosophila* model of mucolipidosis type IV due to defective clearance of apoptotic cells. Cell **135**, 838–851 (2008). 19041749 10.1016/j.cell.2008.09.041 PMC2649760

[18] W. D. Tracey, R. I. Wilson, G. Laurent, S. Benzer, *painless*, a *Drosophila* gene essential for nociception. Cell **113**, 261–273 (2003). 12705873 10.1016/s0092-8674(03)00272-1

[19] B. Al-Anzi, W. D. Tracey Jr., S. Benzer, Response of *Drosophila* to wasabi is mediated by *painless*, the fly homolog of mammalian TRPA1/ANKTM1. Curr. Biol. **16**, 1034–1040 (2006). 16647259 10.1016/j.cub.2006.04.002

[20] S. J. Mandel, M. L. Shoaf, J. T. Braco, W. L. Silver, E. C. Johnson, Behavioral aversion to AITC requires both *painless* and *dTRPA1* in *Drosophila*. Front. Neural Circuits **12**, 45 (2018). 30018539 10.3389/fncir.2018.00045 PMC6038230

[21] T. Sokabe, S. Tsujiuchi, T. Kadowaki, M. Tominaga, *Drosophila* painless is a Ca^2+^-requiring channel activated by noxious heat. J. Neurosci. **28**, 9929–9938 (2008). 18829951 10.1523/JNEUROSCI.2757-08.2008 PMC6671277

[22] M. E. De Obaldia , Differential mosquito attraction to humans is associated with skin-derived carboxylic acid levels. Cell **185**, 4099–4116 (2022). 36261039 10.1016/j.cell.2022.09.034 PMC10069481

[23] J. J. Zhu , Better than DEET repellent compounds derived from coconut oil. Sci. Rep. **8**, 14053 (2018). 30232355 10.1038/s41598-018-32373-7 PMC6145915

[24] A. M. Jones , Isolation and identification of mosquito (*Aedes aegypti*) biting deterrent fatty acids from male inflorescences of breadfruit (*Artocarpus altilis* (Parkinson) Fosberg). J. Agric. Food Chem. **60**, 3867–3873 (2012). 22420541 10.1021/jf300101w

[25] C. L. Cantrell , Biting deterrency of undecanoic acid and dodecanoic acid ester analogs against *Aedes aegypti*. Pest. Manag. Sci. **77**, 3737–3743 (2020). 32648638 10.1002/ps.5994

[26] A. Ali , *Aedes aegypti* (Diptera: Culicidae) biting deterrence: Structure-activity relationship of saturated and unsaturated fatty acids. J. Med. Entomol. **49**, 1370–1378 (2012). 23270165 10.1603/me12026

[27] P. Masek, A. C. Keene, *Drosophila* fatty acid taste signals through the PLC pathway in sugar-sensing neurons. PLoS Genet. **9**, e1003710 (2013). 24068941 10.1371/journal.pgen.1003710 PMC3772025

[28] H. Kim , *Drosophila* *Gr64e* mediates fatty acid sensing via the phospholipase C pathway. PLoS Genet. **14**, e1007229 (2018). 29420533 10.1371/journal.pgen.1007229 PMC5821400

[29] J. M. Tauber , A subset of sweet-sensing neurons identified by IR56d are necessary and sufficient for fatty acid taste. PLoS Genet. **13**, e1007059 (2017). 29121639 10.1371/journal.pgen.1007059 PMC5697886

[30] J. E. Ahn, Y. Chen, H. Amrein, Molecular basis of fatty acid taste in *Drosophila*. eLife **6**, e30115 (2017). 29231818 10.7554/eLife.30115 PMC5747521

[31] L. Legal, B. Chappe, J. M. Jallon, Molecular basis of *Morinda citrifolia* (L.): Toxicity on *Drosophila*. J. Chem. Ecol. **20**, 1931–1943 (1994). 24242720 10.1007/BF02066234

[32] R. N. Pradhan, B. Shrestha, Y. Lee, Molecular basis of hexanoic acid taste in *Drosophila melanogaster*. Mol. Cells **46**, 451–460 (2023). 37202372 10.14348/molcells.2023.0035 PMC10336273

[33] M. Dey, E. Brown, S. Charlu, A. Keene, A. Dahanukar, Evolution of fatty acid taste in drosophilids. Cell Rep. **42**, 113297 (2023). 37864792 10.1016/j.celrep.2023.113297 PMC10697176

[34] J. A. Sánchez-Alcañiz , An expression atlas of variant ionotropic glutamate receptors identifies a molecular basis of carbonation sensing. Nat. Commun. **9**, 4252 (2018). 30315166 10.1038/s41467-018-06453-1 PMC6185939

[35] Y. Qian , Identification of transient receptor potential channel genes from the swimming crab, *Portunus trituberculatus*, and their expression profiles under acute temperature stress. BMC Genomics **25**, 72 (2024). 38233779 10.1186/s12864-024-09973-x PMC10795286

[36] M. T. Kozma , Chemoreceptor proteins in the Caribbean spiny lobster, *Panulirus argus*: Expression of Ionotropic Receptors, Gustatory Receptors, and TRP channels in two chemosensory organs and brain. PLoS One **13**, e0203935 (2018). 30240423 10.1371/journal.pone.0203935 PMC6150509

[37] A. Abramova, M. Alm Rosenblad, A. Blomberg, T. A. Larsson, Sensory receptor repertoire in cyprid antennules of the barnacle *Balanus improvisus*. PLoS One **14**, e0216294 (2019). 31048879 10.1371/journal.pone.0216294 PMC6497305

[38] M. T. Kozma , Comparison of transcriptomes from two chemosensory organs in four decapod crustaceans reveals hundreds of candidate chemoreceptor proteins. PLoS One **15**, e0230266 (2020). 32163507 10.1371/journal.pone.0230266 PMC7067487

[39] T. Shiraiwa, J. R. Carlson, Proboscis extension response (PER) assay in Drosophila. J. Vis. Exp. **3**, 193 (2007). 10.3791/3193. PMC2535836 18978998

[40] J. Liu , Alleviation of thermal nociception depends on heat-sensitive neurons and a TRP channel in the brain. Curr. Biol. **33**, 2397–2406.e2396 (2023). 37201520 10.1016/j.cub.2023.04.055 PMC10330845

[41] D. A. Gorczyca , Identification of Ppk26, a DEG/ENaC channel functioning with Ppk1 in a mutually dependent manner to guide locomotion behavior in *Drosophila*. Cell Rep. **9**, 1446–1458 (2014). 25456135 10.1016/j.celrep.2014.10.034 PMC4254518

[42] Y. Y. Wang, R. B. Chang, S. D. Allgood, W. L. Silver, E. R. Liman, A TRPA1-dependent mechanism for the pungent sensation of weak acids. J. Gen. Physiol. **137**, 493–505 (2011). 21576376 10.1085/jgp.201110615 PMC3105510

[43] Y. Kwon, H. S. Shim, X. Wang, C. Montell, Control of thermotactic behavior via coupling of a TRP channel to a phospholipase C signaling cascade. Nat. Neurosci. **11**, 871–873 (2008). 18660806 10.1038/nn.2170

[44] E. S. Hodgson, J. Y. Lettvin, K. D. Roeder, Physiology of a primary chemoreceptor unit. Science **122**, 417–418 (1955). 13246649 10.1126/science.122.3166.417-a

[45] R. Delventhal, A. Kiely, J. R. Carlson, Electrophysiological recording from Drosophila labellar taste sensilla. J. Vis. Exp. **85**, e51355. (2014). 10.3791/51355 PMC4089476 24638081

[46] N. Fujishiro, H. Kijima, H. Morita, Impulse frequency and action potential amplitude in labellar chemosensory neurons of *Drosophila melanogaster*. J. Insect Physiol. **30**, 317–325 (1984).

[47] R. Y. Hwang, N. A. Stearns, W. D. Tracey, The ankyrin repeat domain of the TRPA protein Painless is important for thermal nociception but not mechanical nociception. PLoS One **7**, e30090 (2012). 22295071 10.1371/journal.pone.0030090 PMC3266251

[48] B. J. Matthews, C. S. McBride, M. DeGennaro, O. Despo, L. B. Vosshall, The neurotranscriptome of the *Aedes aegypti* mosquito. BMC Genomics **17**, 32 (2016). 26738925 10.1186/s12864-015-2239-0 PMC4704297

[49] M. Li , Germline Cas9 expression yields highly efficient genome engineering in a major worldwide disease vector, *Aedes aegypti*. Proc. Natl. Acad. Sci. U.S.A. **114**, E10540–E10549 (2017). 29138316 10.1073/pnas.1711538114 PMC5724270

[50] S. Shankar , Optimized genetic tools for neuroanatomical and functional mapping of the *Aedes aegypti* olfactory system. G3 (Bethesda) **15**, jkae307 (2025). 39853276 10.1093/g3journal/jkae307 PMC11917485

[51] D. Feir, J. I. Lengy, W. B. Owen, Contact chemoreception in the mosquito, *Culiseta-Inornata* (Williston)—Sensitivity of the tarsi and labella to sucrose and glucose. J. Insect Physiol. **6**, 13–20 (1961).

[52] E. H. Super, Sensory hairs with permeable tips on the tarsi of the yellow-fever mosquito, *Aedes aegypti*. Ann. Entomol. Soc. Am. **55**, 531–535 (1962).

[53] E. J. Dennis, O. V. Goldman, L. B. Vosshall, *Aedes aegypti* mosquitoes use their legs to sense DEET on contact. Curr. Biol. **29**, 1551–1556.e55 (2019). 31031114 10.1016/j.cub.2019.04.004 PMC6504582

[54] B. H. King, P. B. Gunathunga, Gustation in insects: Taste qualities and types of evidence used to show taste function of specific body parts. J. Insect Sci. **23**, 11 (2023), 10.1093/jisesa/iead1018. PMC10072106 37014302

[55] F. Ling, A. Dahanukar, L. A. Weiss, J. Y. Kwon, J. R. Carlson, The molecular and cellular basis of taste coding in the legs of *Drosophila*. J. Neurosci. **34**, 7148–7164 (2014). 24849350 10.1523/JNEUROSCI.0649-14.2014 PMC4028494

[56] Y. F. Chang, J. S. Imam, M. F. Wilkinson, The nonsense-mediated decay RNA surveillance pathway. Annu. Rev. Biochem. **76**, 51–74 (2007). 17352659 10.1146/annurev.biochem.76.050106.093909

[57] E. B. Brown , Ir56d-dependent fatty acid responses in *Drosophila* uncover taste discrimination between different classes of fatty acids. eLife **10**, e67878 (2021). 33949306 10.7554/eLife.67878 PMC8169106

[58] S. D. Rodriguez, L. L. Drake, D. P. Price, J. I. Hammond, I. A. Hansen, The efficacy of some commercially available insect repellents for *Aedes aegypti* (Diptera: Culicidae) and *Aedes albopictus* (Diptera: Culicidae). J. Insect Sci. **15**, 140 (2015). 26443777 10.1093/jisesa/iev125 PMC4667684

[59] M. T. Gillies, The role of carbon-dioxide in host-finding by mosquitos (Diptera, Culicidae)—A review. Bull. Entomol. Res. **70**, 525–532 (1980).

[60] O. J. Bosch, M. Geier, J. Boeckh, Contribution of fatty acids to olfactory host finding of female *Aedes aegypti*. Chem. Senses **25**, 323–330 (2000). 10866990 10.1093/oxfordjournals.chemse.a014042

[61] S. R. Shanbhag, S. K. Park, C. W. Pikielny, R. A. Steinbrecht, Gustatory organs of *Drosophila melanogaster*: Fine structure and expression of the putative odorant-binding protein PBPRP2. Cell Tissue Res. **304**, 423–437 (2001). 11456419 10.1007/s004410100388

[62] M. Hiroi, F. Marion-Poll, T. Tanimura, Differentiated response to sugars among labellar chemosensilla in *Drosophila*. Zool. Sci. **19**, 1009–1018 (2002). 10.2108/zsj.19.1009 12362054

[63] S. R. Hill, J. J. B. Smith, Consistent pattern in the placement of taste sensilla on the labellar lobes of. Int. J. Insect Morphol. **28**, 281–290 (1999).

[64] I. Kimura, A. Ichimura, R. Ohue-Kitano, M. Igarashi, Free fatty acid receptors in health and disease. Physiol. Rev. **100**, 171–210 (2020). 31487233 10.1152/physrev.00041.2018

[65] H. Iikura , Mosquito repellence induced by tarsal contact with hydrophobic liquids. Sci. Rep. **10**, 14480 (2020). 32879341 10.1038/s41598-020-71406-y PMC7468126

[66] S. Dhakal, A. E. Bontempo, R. Singh, P. Dhavan, C. Montell, Gustatory avoidance of fatty acids by Aedes aegypti depends on an arthropod-specific TRP channel. Dryad 10.5061/dryad.qrfj6q5ww. Deposited 20 January 2026. PMC12912979 41662530

