# Supplementary material for: Gustatory avoidance of fatty acids by Aedes aegypti depends on an arthropod-specific TRP channel
Source: Proc Natl Acad Sci U S A. 2026 Feb 9;123(7):e2522818123. doi: 10.1073/pnas.2522818123 (PMC12912979; doi:10.1073/pnas.2522818123)
Supplement: Supplementary file 1 — Appendix 01 (PDF) [file pnas.2522818123.sapp.pdf]

Supplementary Information for

Gustatory avoidance of fatty acids by *Aedes aegypti* depends on an arthropod-specific TRP channel

Subash Dhakal*, Angela E. Bontempo*, Ramandeep Singh,

Pratik Dhavan, Craig Montell[2,]**

[2]Lead Contact

*These authors contributed equally.
**Corresponding author. Email: cmontell@ucsb.edu

This PDF file includes:

Figures S1 to S9
Legends for S1 to S9

Supplementary Table 1 and Table 2
Legends for Supplementary Table 1 and Table 2
Legend for Supplementary Video 1
Supplementary Materials and Methods
Supplementary references

Supplementary Figure 1

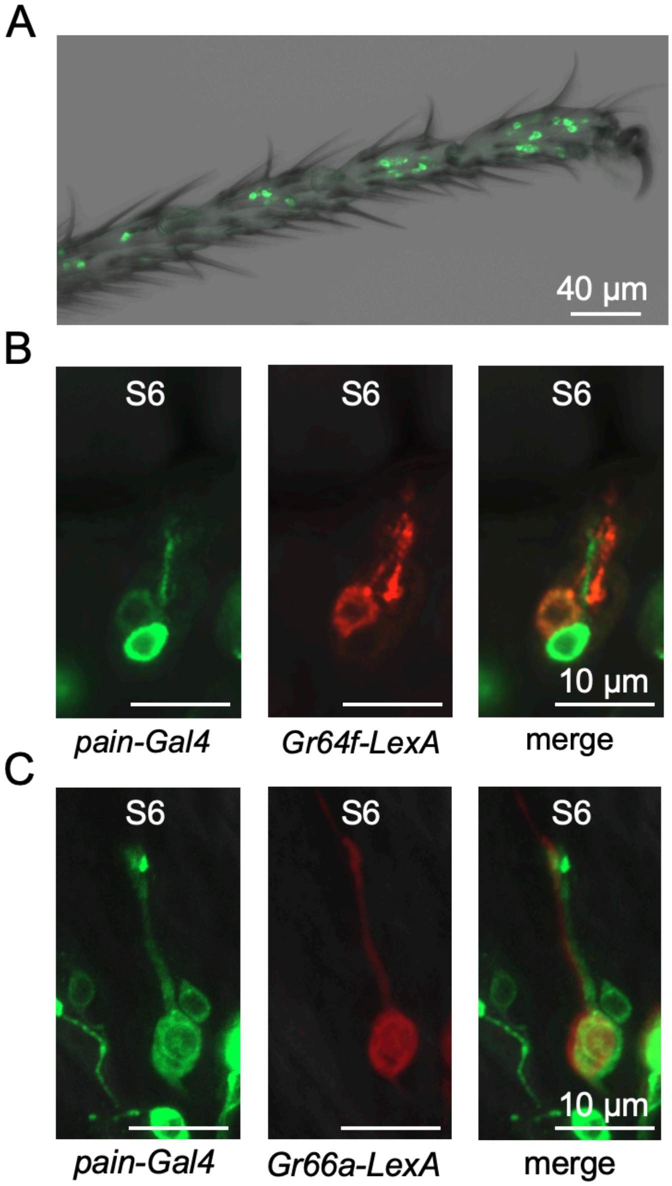

Fig. S1. *Drosophila pain* reporter expression in foreleg tarsi and the A and B GRNs of S6 sensilla. (*A*) Image of *pain-Gal4* driving *40xUAS-IVS-mCD8::GFP* in tarsal segments of a *Drosophila* foreleg. Scale bar, 40 µm. n=5. **(***B***,** *C)* Reporter expression in S6 sensilla. Scale bars, 10 µm. (*B*) Left panel, *pain-Gal4* driving *40xUAS-IVS-mCD8::GFP*. Middle panel, *Gr64f-LexA* driving *LexAop-rCD2::RFP*. Right panel, merge. (*C*) Left panel, *pain-Gal4* driving *40xUAS-IVS-mCD8::GFP*. Middle panel, *Gr66a-LexA* driving *13xLexAop-6xmCherry.* Right panel, merge.

Supplementary Figure 2

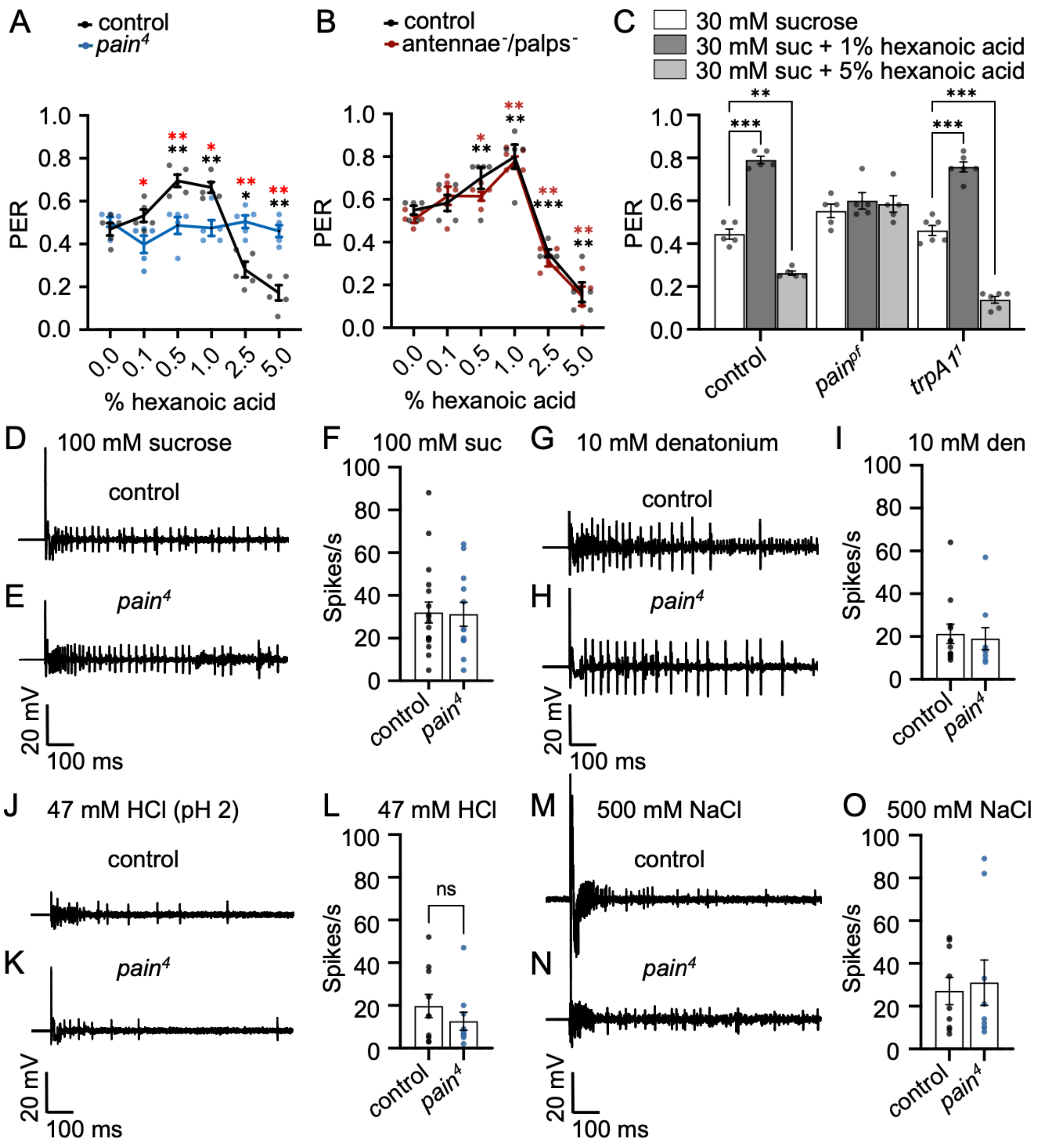

Fig. S2. *Drosophila pain* mutants display gustatory defects in response to hexanoic acid (HA) but not to other compounds that activate GRNs in S6. (*A-C*) PER assays performed by stimulating flies with 30 mM sucrose to establish a baseline response, and then with 30 mM sucrose mixed with HA. (*A*) PER assays performed by stimulating foreleg tarsi with 30 mM sucrose alone or using 30 mM sucrose mixed with 0.1-5% HA. The red asterisks indicate significant differences between the control and *pain*^{4}, and the

black asterisks indicate significant differences between control responses to 30 mM sucrose vs control responses to 30 mM sucrose + % HA. (*B*) PERs to different concentrations of HA using either intact control flies or flies with surgically removed antennae and maxillary palps (antennae^{-}/palps^{-}). The black asterisks indicate significant differences exhibited by intact control flies presented with 30 mM sucrose alone versus 30 mM sucrose and the indicated % HA. The red asterisks indicate significant differences exhibited by flies missing antennae and maxillary palps presented with 30 mM sucrose alone versus 30 mM sucrose and % HA. (*C)* PER assays performed by stimulating labella with 30 mM sucrose alone, or using 30 mM sucrose mixed with 1% or 5% HA. The asterisks indicate significant differences between responses to 30 mM sucrose versus 30 mM sucrose and HA. n=5-6, N=10-12 flies/assay. Concentration-dependent changes in mean PER responses (panels *A*-*C*) were analyzed with repeated measures using one-way ANOVA followed by Dunnett's multiple-comparisons post hoc tests. Residuals were checked for normality with Shapiro-Wilk tests and Geisser-Greenhouse corrections were made. (*D*-*O*) Tip recordings on S6 sensilla. (*D*) Representative trace using 100 mM sucrose on control flies. (*E*) Representative trace using 100 mM sucrose on the *pain*^{4} mutant. (*F*) Average spikes/second using 100 mM sucrose (suc). (*G*) Representative trace using 10 mM denatonium on control flies. (*H*) Representative trace using 10 mM denatonium on the *pain*^{4} mutant. (*I*) Average spikes/second using 10 mM denatonium. (*J*) Representative trace using 47 mM HCl (pH 2) on control flies. (*K*) Representative trace using 47 mM HCl (pH 2) on the *pain*^{4} mutant. (*L*) Average spikes/second using 47 mM HCl (pH 2). (*M*) Representative trace using 500 mM NaCl on control flies. (*N*) Representative trace using 500 mM NaCl on the *pain*^{4} mutant. (*O*) Average spikes/second using 500 mM NaCl. When quantifying spikes/second, the first 50 ms are excluded due to contact artifacts. n=10-12. For differences between genotypes, Mann-Whitney U tests were used. Error bars, SEMs. One asterisk, $P < 0.05$. Two asterisks, $P < 0.01$. Three asterisks, $P < 0.001$.

Supplementary Figure 3

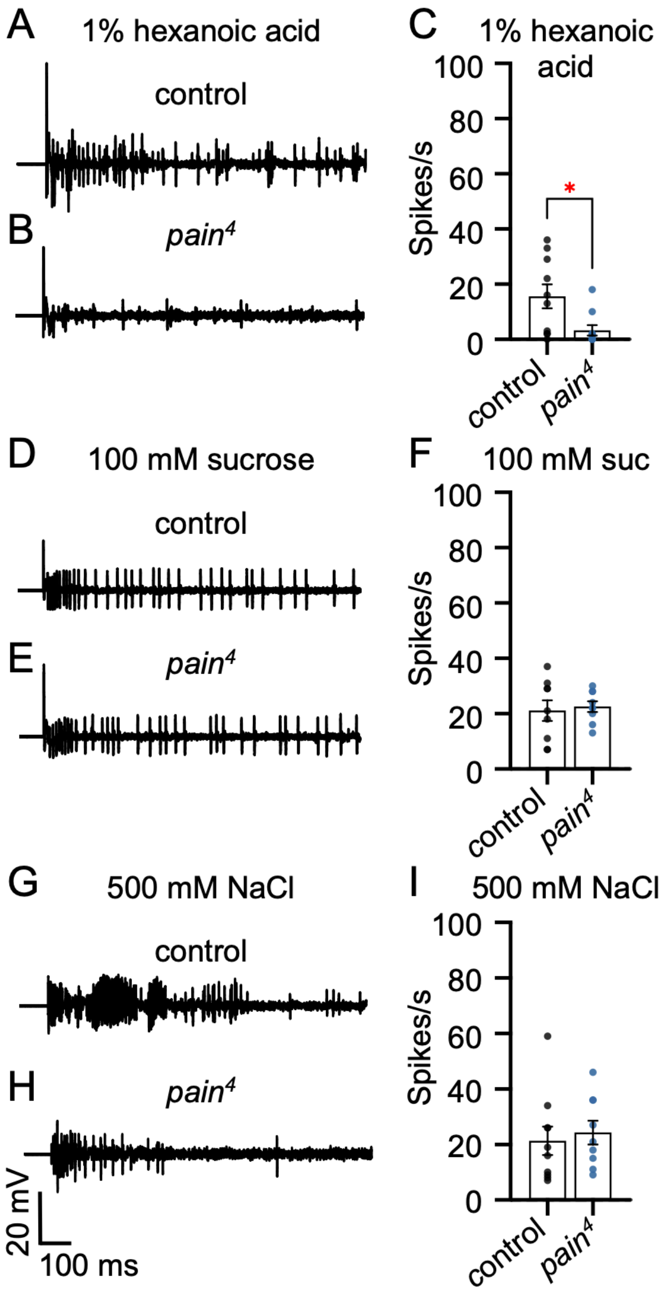

Fig. S3 Tip recordings demonstrating that GRNs in L4 sensilla from *Drosophila pain^4* mutants display reduced action potential frequencies in response to HA but not to other compounds. (*A*) Representative trace from control flies in response to 1% HA. (*B*) Representative trace from *pain^4* flies in response to 1% HA. (*C*) Average spikes/second using 1% HA. (*D*) Representative trace from control flies in response to 100 mM sucrose. (*E*) Representative trace from *pain^4* flies in response to 100 mM sucrose. (*F*) Average spikes/second using 100 mM sucrose. (*G*) Representative trace from control flies in response to 500 mM NaCl. (*H*) Representative trace from *pain^4* flies in response to 500 mM NaCl. (*I*) Average spikes/second using 500 mM NaCl. n=9-11. Mann-

Whitney U tests were used to determine differences between genotypes. Error bars, SEMs. $^{*}P < 0.05$.

Supplementary Figure 4

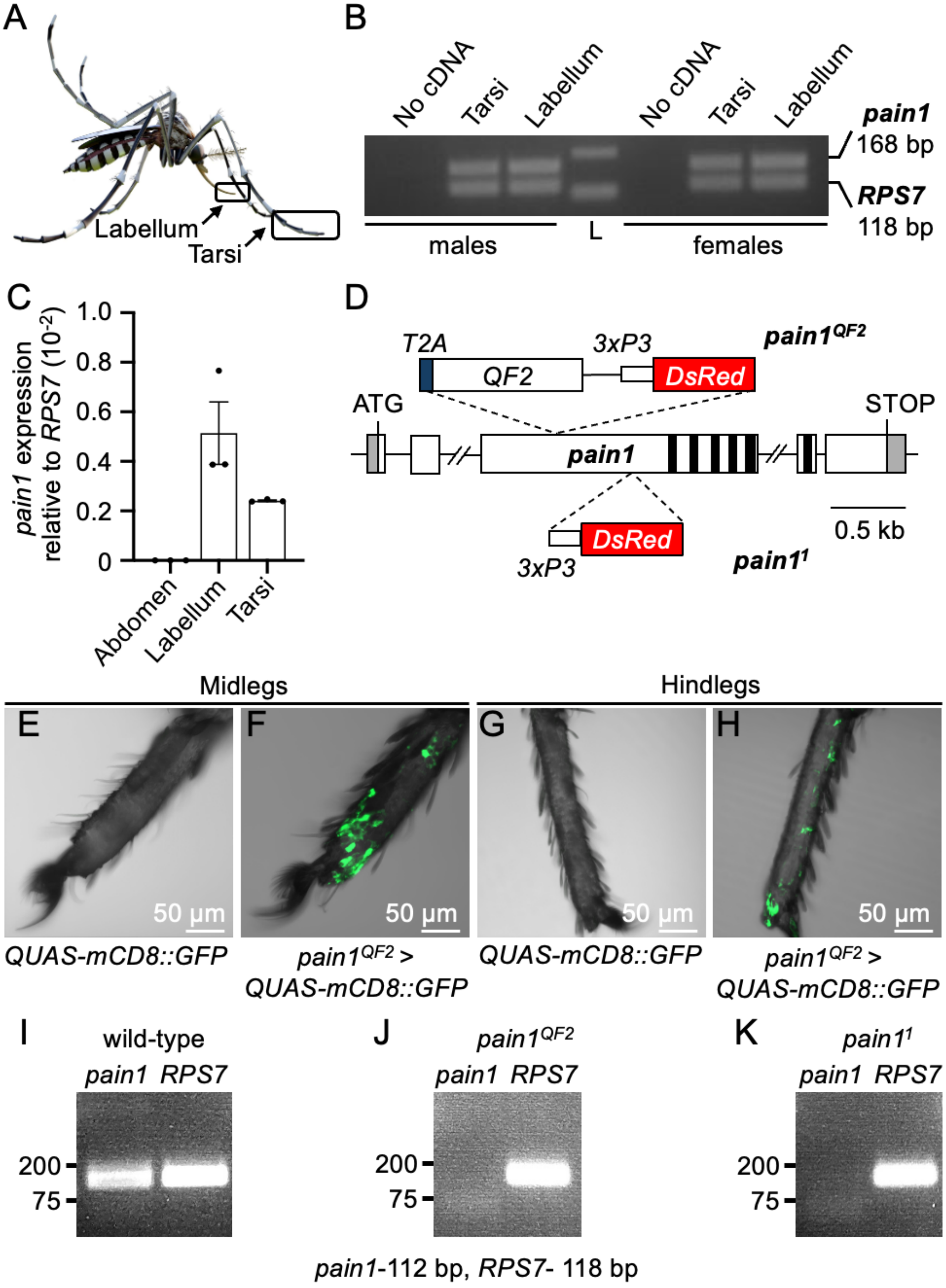

Fig. S4. Expression of *Ae. aegypti pain1* in gustatory organs. (*A*) Cartoon of an *Ae. aegypti* female with the labellum and a foreleg tarsi indicated. (*B*) 2.0% agarose gel showing the *pain1* RT-PCR product (168 bp) using cDNAs prepared from RNA extracted from the labella and forelegs from males and females. The *ribosomal protein S7* (*RPS7)* PCR product was amplified and used as an internal control. "L" indicates the DNA size markers (75 and 200 bp). (*C*) RT-qPCR showing expression of *pain1* relative to *RPS7* in the abdomens, proboscises, and

forelegs tarsi from LVP females. n=3. (*D*) Cartoon showing the *pain1*^{QF2} (*pain1-T2A-QF2*) and *pain1*^{1} alleles generated using CRISPR-Cas9. (*E-H*) *pain1*^{QF2} reporter expression in the most distal tarsi of the midlegs and hindlegs from females. To reduce the color of the cuticle and improve detection of the reporter, *pain1*^{QF2} was used to drive expression of *QUAS*-m*CD8*::*GFP* in a *yellow* mutant background. GFP was detected by staining with anti-GFP (green). The images were acquired using a Zeiss LSM 900 confocal microscope. The scale bars represent 50 µm. (*E*) Distal tarsal segment from a midleg of a *QUAS*-m*CD8*::*GFP* female. (*F*) *pain1*^{QF2}>*QUAS*-m*CD8*::*GFP* distal tarsal segment from a midleg. (*G*) Distal tarsal segment from a hindleg of a *QUAS*-m*CD8*::*GFP* female. (*H*) Distal tarsus from a hindleg of a *pain1*^{QF2}>*QUAS*-m*CD8*::*GFP* female. (*I-K*) Confirmation of the *pain1*^{1} and *pain1*^{QF2} alleles by RT-PCR. The cDNAs were prepared from whole bodies of female *Ae. aegypti* of the indicated genotypes. The *pain1* and *ribosomal protein S7* (*RPS7*) PCR products are 112 bp and 118 bp, respectively. (*I*) Wild-type. (*J*) *pain1*^{QF2}. (*K*) *pain1*^{1}.

Supplementary Figure 5

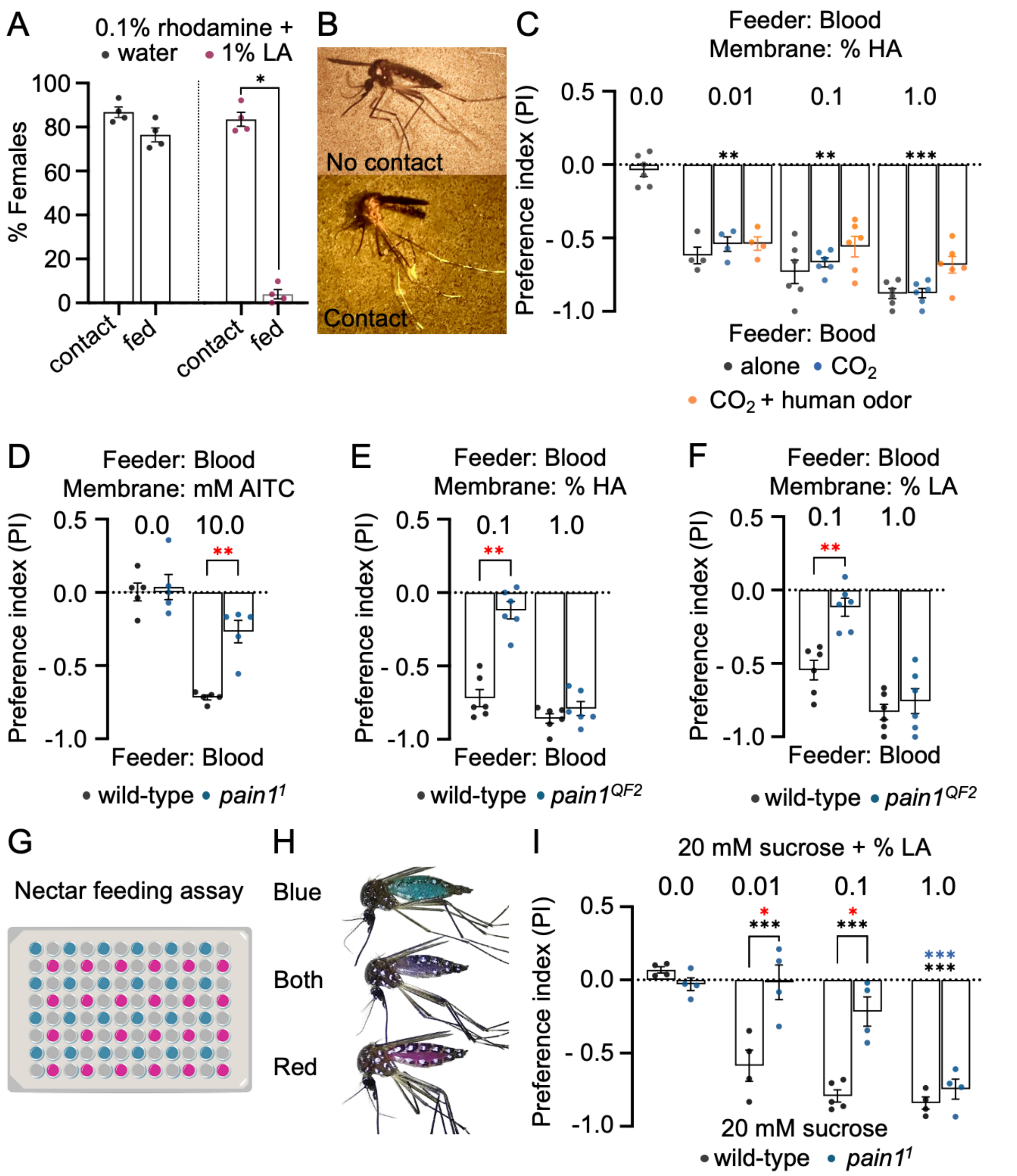

Fig. S5. *Ae. aegypti pain1* mutants exhibit defects in FA taste in the context of either blood feeding or sugar feeding. (*A*) Single blood feeding assay with membranes coated in 0.1% rhodamine B dye plus either water plus solvent (8% ethanol) or 1% linoleic acid (LA) in the solvent (8% ethanol). The percentage of wild-type females that landed on the feeder (contact) and the percentage that successfully engorged (fed) were quantified.

n=4. N=50 mosquitoes/assay. (*B*) Representative images of females that did not make contact with the dye-soaked membrane (top) or did make contact (bottom). (*C*) Testing wild-type using blood feeders with one membrane soaked in the indicated percentage of hexanoic acid (HA) plus solvent and the other membrane soaked in water plus solvent. In the indicated experiments, both blood feeders were exposed to 5% CO_2, and in other selected assays both blood feeders were exposed to 5% CO_2 and human odor from a worn nitrile glove. The blood in each of the two feeders contained either 0.1% rhodamine B (red) or 0.1% fluorescein (green) n≥4. N=50 mosquitoes/assay. (*D*) Testing wild-type and *pain1*^{1} using blood feeders with one membrane soaked in 10 mM AITC plus solvent (1% DMSO) and the other membrane soaked in water plus the solvent. n=5. N=50 mosquitoes/assay. (*E, F*) Two-way choice blood feeder assays using wild-type and *pain1*^{QF2} females. The two blood feeder membranes used in each assay were laced either with the indicated FAs (0.1% or 1%) plus solvent or soaked in water plus solvent. The blood in each of the two feeders contained either 0.1% rhodamine B (red) or 0.1% fluorescein (green). n=6. N=50 mosquitoes/assay. (*E*) 0.1 or 1% HA versus water. (*F*) 0.1 or 1% LA versus water. n=6. (*G*) Image of a 96-well plate used for the two-way nectar feeding assay. The two food options contained either 0.09 mg/mL sulforhodamine B (red) or 0.09 mg/mL brilliant blue FCF. (*H*) Images of adult females after a nectar feeding assay obtained using a stereomicroscope (AmScope SW-2B). A purple abdomen (both) indicates consumption of both red and blue foods. (*I*) Two-way nectar feeding assays with wild-type and *pain1*^{1} females. Mosquitoes were allowed to choose between 20 mM sucrose plus 8% ethanol or 20 mM sucrose, 8% ethanol, laced with the indicated percentages of LA for 3 hrs. n=4-5, N= 50 mosquitoes/assay. The black asterisks indicate significant differences between wild-type choosing 0% versus 0.01%, 0.1%, and 1.0% LA. The blue asterisks indicate significant differences between *pain1*^{1} choosing 0% versus 0.01%, 0.1%, and 1.0% LA. The red asterisks indicate significant differences between wild-type and *pain1*^{1}. The statistics within each genotype for different conditions were determined with one-way ANOVA followed by Dunnett’s multiple-comparisons post hoc tests. Residuals were tested for normality using Shapiro-Wilk tests and for equal variances with the Brown-Forsythe tests (panels *A-E, H*). For differences between each genotype, Mann-Whitney U tests were used. Error bars, SEMs. One asterisk, $P < 0.05$. Two asterisks, $P < 0.01$. Three asterisks, $P < 0.001$.

Supplementary Figure 6

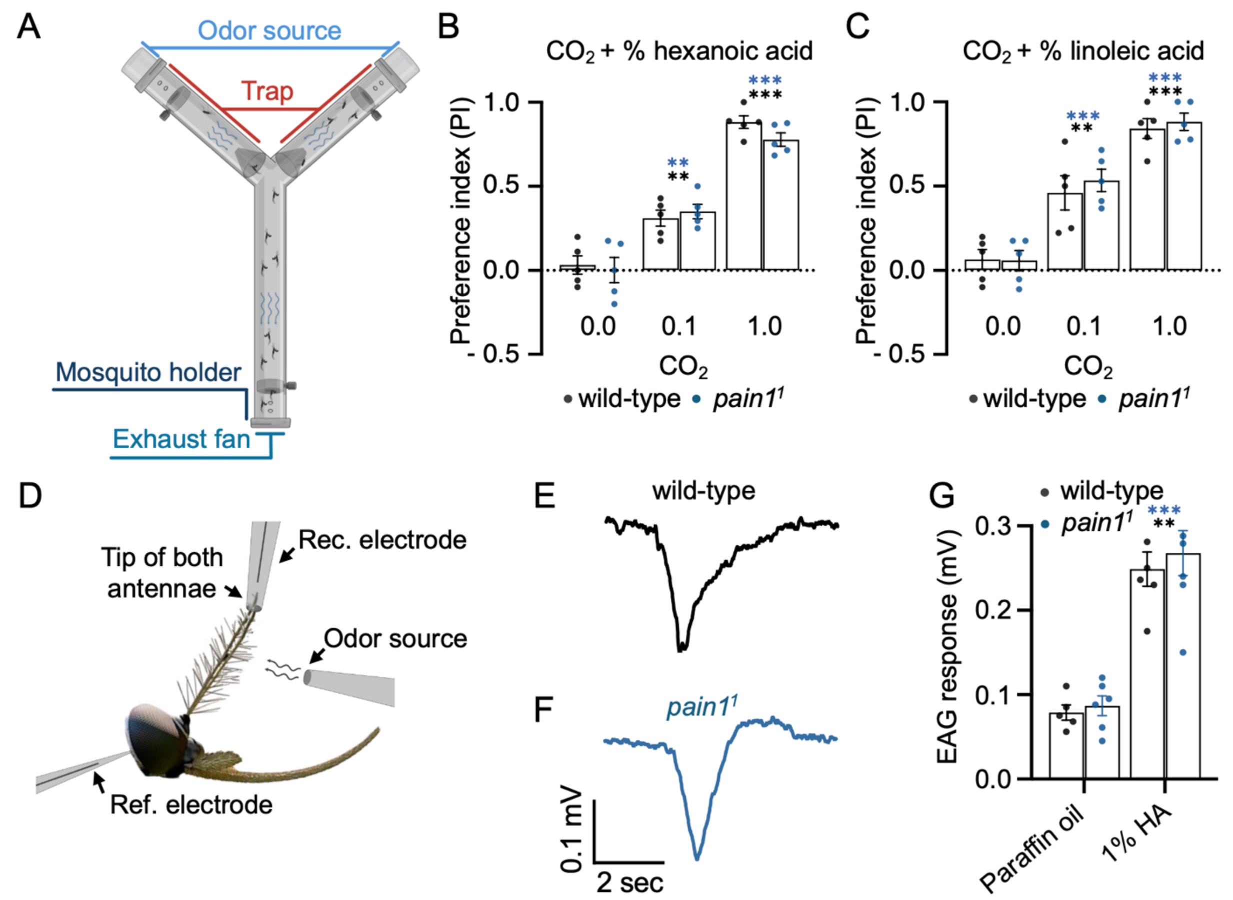

Fig. S6. *Ae. aegypti pain1* mutants display normal olfactory behavior towards FAs. (*A*) Cartoon of the Y-tube olfactometer used for the olfactory assays, similar to a previously described design (1). An electric exhaust fan was attached at the bottom of the tube. The sources of the odors, the mosquito trap zones, and the mosquito holding area are indicated. (*B, C*) 20 females were used per olfactory Y-tube assay. Wild-type and *pain1*[1] mosquitoes were given a choice between 5% CO_2 only versus 5% CO_2 plus the indicated concentrations of FAs. n=5. (*B*) Preference indexes after mosquitoes were given a choice between 5% CO_2 versus 5% CO_2 plus 0.1% or 1% hexanoic acid. (*C*) Preference indexes after mosquitoes were given a choice between 5% CO_2 versus 5% CO_2 plus 0.1% or 1% linoleic acid. (*D*) Cartoon depicting the setup for performing EAGs on *Ae. aegypti* females. (*E*) Sample wild-type EAG trace in response to 1% HA. (*F*) Sample *pain1*[1] EAG trace in response to 1% HA. (*G*) Quantification of EAG responses (mV) obtained from wild-type and *pain1*[1] with paraffin oil (control) and 1% HA. n=5-6. The statistics within each genotype for different conditions were checked with one-way ANOVA followed by Dunnett's multiple-comparisons post hoc test. Residuals were tested for normality using the Shapiro-Wilk test and for equal variances with the Brown-Forsythe test (panels *B-C*). The statistics for EAG responses within genotypes were tested with paired t tests. Datasets were tested for normality using the Shapiro-Wilk test (panel *G*). For differences between each genotype, Mann-Whitney U tests were used.

The black asterisks indicate significant differences between the paraffin oil control and 1% HA for wild-type females. The blue asterisks indicate significant differences between paraffin oil and 1% HA for *pain1*^{1} females. Error bars, SEMs. Two asterisks, $P < 0.01$. Three asterisks, $P < 0.001$.

Supplementary Figure 7

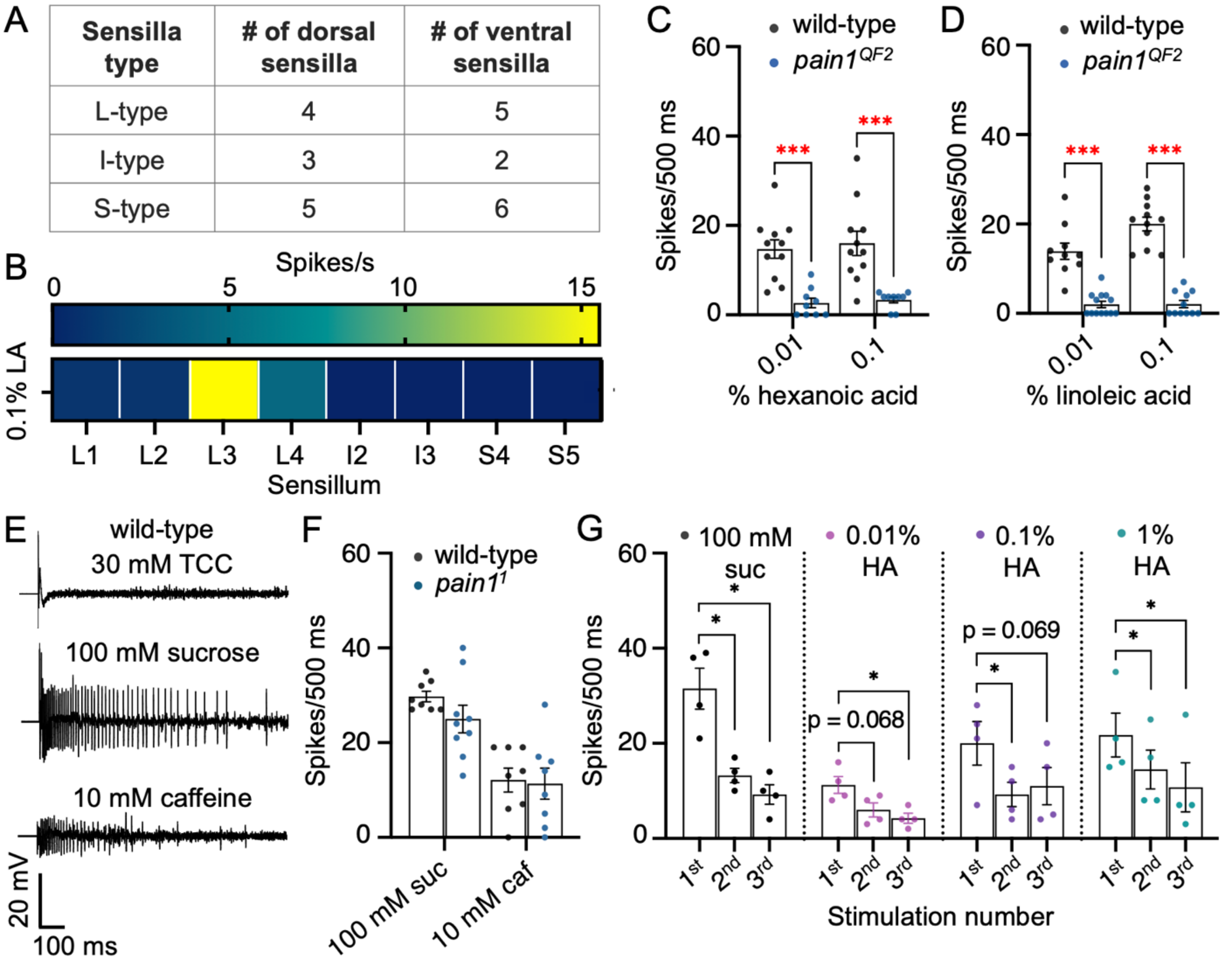

Sensilla type	# of dorsal sensilla	# of ventral sensilla
L-type	4	5
I-type	3	2
S-type	5	6

Fig. S7. Number of gustatory sensilla on the labellum, and tip recordings with FAs using the *pain1*^{QF2} allele. (*A*) Number of L-type, I-type, and S-type sensilla housed on the dorsal and ventral sides of each of the two bilaterally-symmetrical labella from *Ae. aegypti* females. (*B-D*) Tip recordings showing the frequencies of action potentials upon application of the indicated FAs to gustatory sensilla located on the dorsal side of female labella. (*B*) Heat map showing average frequencies of action potentials in response to 0.1% LA from the indicated sensilla. (*C*) Spikes/500 ms obtained from wild-type and *pain1*^{QF2} using the indicated concentrations of HA. (*D*) Spikes/500 ms obtained from wild-type and *pain1*^{QF2} with the indicated concentrations of LA. (*E*) Representative traces obtained with 30 mM TCC, 100 mM sucrose and 10 mM caffeine. (*F*) Average spikes/500 ms with 100 mM sucrose (suc) and 10 mM caffeine (caf). (*G*) Frequencies of action potentials upon application of the indicated concentrations of sucrose or HA to L3 gustatory sensilla located on the dorsal side of female labella. Spikes/500 ms were obtained from wild-type females. Three consecutive stimuli were delivered at 3 sec intervals to assess adaptation. Asterisks indicate significant differences from the controls. n=4-11. For differences between each genotype, Mann-Whitney U tests were used. To compare the frequencies of action potentials between the first versus either

the second or third stimulations with either sucrose or HA (*G*), we employed paired Student's *t*-tests. Error bars, SEMs. One asterisk, $P < 0.05$. Three asterisks, $P < 0.001$.

Supplementary Figure 8

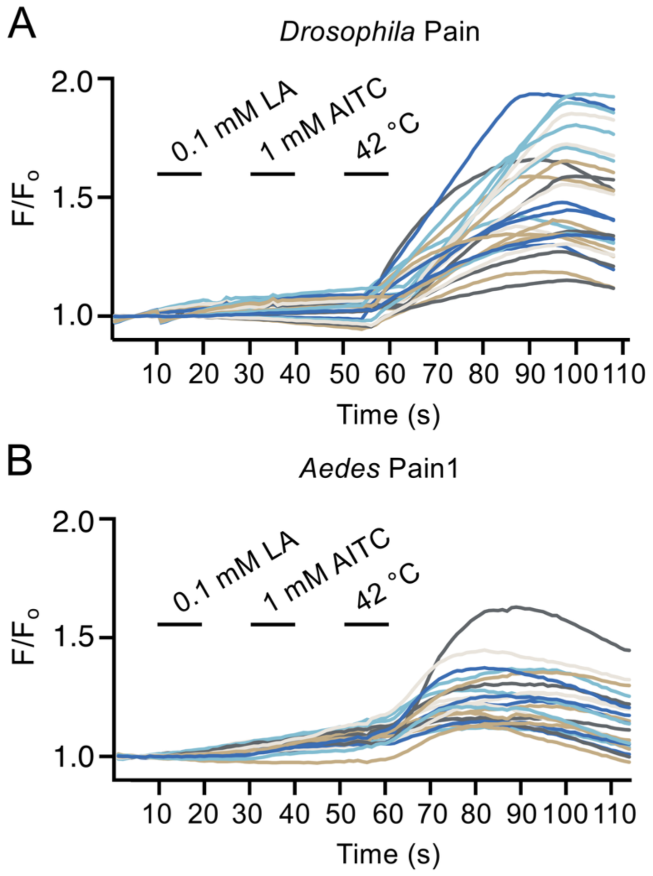

Fig. S8. Ca^{2+}photometry to test whether LA activates *Drosophila* Pain or *Aedes* Pain1 expressed *in vitro*. Pain and Pain1 were expressed in HEK293 cells. The cells were loaded with Fluo-8 to assay free Ca^{2+} levels before (F_o) and after stimulation (F), with 100 µM LA, 1 mM AITC, and 42 °C. F/F_o was then determined. (*A*) *Drosophila* Pain. (*B*) *Aedes* Pain1. n=3 independent experiments.

Supplementary Figure 9

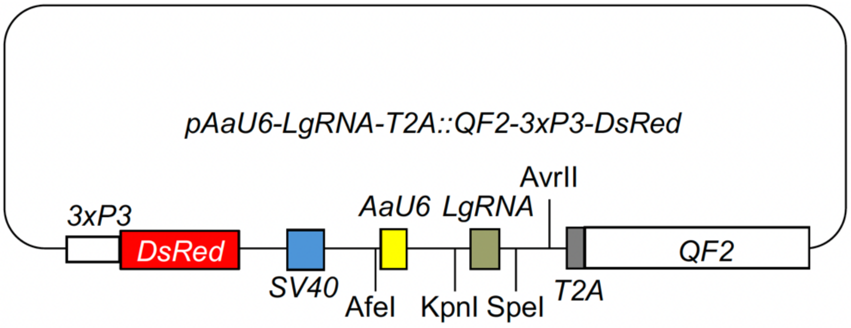

Fig. S9. Vector for generating *pain1*^{QF2}. Cartoon of the *pAaU6-LgRNA-T2A::QF2-3xP3-DsRed* vector, which is engineered to enable insertion of *T2A::QF2* in *Ae. aegypti*. The vector includes the *T2A::QF2* gene, and *DsRed* driven by the *3xP3* promoter. Transcription is stopped using the *SV40* transcriptional terminator. The vector also includes the *U6* promoter and the *LgRNA* scaffold sequence for expressing gRNAs. To facilitate subcloning the upstream and downstream homology arms, AvrII and AfeI restriction enzyme sites are included, respectively. KpnI and SpeI sites are included for inserting single guide RNAs (sgRNAs).

Supplementary Table 1. List of oligonucleotides sequences related to *Drosophila* methods.

**Oligonucleotides**	**Source**
P1: 5'-GTTGTCGTGACTTCATTGAAGC-3'	This paper
P2: 5'-ACCAGGAGGAATACTTCGG-3'	This paper
P3: 5'-ACCGCCATACCAGTATCTAC-3'	This paper
P4: 5'-GCCGTCTCTACGAGATTGG-3'	This paper
P5: 5'-TTAGTCGTTGTCTGGATATTAACGA -3'	This paper
P6: 5'-ATTTTCCTGCTCTTCGTGATC -3'	This paper
P7: 5'-TCACTTCCGGTCCTGGA-3'	This paper
rp49 RT-PCR primers:<br>F: 5'-TGCTAAGCTGTCG-3'<br>R: 5'-TCTCCTTGCGCTTCTTGGAG-3'	(2)

Supplementary Table 2. List of oligonucleotides sequences related to *Aedes* methods.

**Oligonucleotides**	**Source**
pain1^{1} sgRNA sequence:<br>5'-GAATACAAATCTAATTACAC-3'	This paper
pain1^{QF2} sgRNA sequence:<br>5'-CTCAACAGGACTCATCCGCA-3'	This paper
pain1^{1} upstream homology arm:<br>F: 5'- ATCTGATCTTCGAGGTGATCGAC-3'<br>R: 3'-TAATTAGATTTGTATTCCGGTGGAACCAAACAG-5'	This paper
pain1^{1} downstream homology arm:<br>F: 5'-CTAGCACCGGTGATAGAACAGT-3'<br>R: 3'-CAGGGCTGAGGGTCACTACTAC-5'	This paper
pain1^{QF2} upstream homology arm:<br>F: 5'-GCGGTACCTTACGTCAAAACC-3'<br>R: 3'-GGATGAGTCCTGTTGAGTCTTTTCC-5'	This paper
pain1^{QF2} downstream homology arm:<br>F: 5'-GCATGGAAGAAAGATAAAACACAAC-3'<br>R: 3'-CGATCCAACCAGCAGGGTAA-5'	This paper
pain1^{1} genotyping primers:<br>F: 5'-TCGCGACAATGACTTACGACT-3'<br>R: 3'- CTGACCGAGTTCTCTCAGCG-5'	This paper
pain1^{QF2} genotyping primers:<br>F: 5'-TCGCGACAATGACTTACGACT-3'<br>R: 3'- CTGACCGAGTTCTCTCAGCG-5'	This paper
pain1 RT-PCR primers:<br>F: 5'- CGCTTTGAGCTGTGGCGC -3'<br>R: 3'- AAGTGGATGGGATACTGGCC -5'	This paper
Rps7 RT-PCR primers:<br>F: 5'-TCAGTGTACAAGAAGCTGACCGGA-3'<br>R: 3'-TTCCGCGCGCGCTCACTTATTAGATT-5'	This paper
pain1 qRT-PCR primers:<br>F: 5'- CGCTTTGAGCTGTGGCGC -3'<br>R: 3'- AAGTGGATGGGATACTGGCC -5'	This paper
Rps7 qRT-PCR primers:<br>F: 5'-TCAGTGTACAAGAAGCTGACCGGA-3'<br>R: 3'-TTCCGCGCGCGCTCACTTATTAGATT-5'	This paper

Supplementary Video legend

Supplementary Video 1. Slow-motion landing of *Ae. aegypti* females on a blood feeder with 1% linoleic acid (LA). Video footage was recorded at 60 fps using a Canon EOS Rebel T6X using an Altura super macro lens and played back via Clipchamp at 6 fps. The video shows females approaching, landing, and making contact with their tarsi and labellum on the feeders. The video illustrates reduced sustained feeding if LA is present, despite initial landing, consistent with LA-induced gustatory avoidance on contact.

Supplemental Materials and Methods

Drosophila stocks

The following lines were obtained from the Bloomington Drosophila Stock Center: w^{1118} (5905), *40xUAS-IVS-mCD8*::*GFP* (32195), *13xLexAop-6xmCherry* (*52271*)*, UAS-mCD8*::*DsRed* (27398), *UAS-10x-IVS-mCD8::RFP*, *13xLexAop2-mCD8::GFP* (32229), *LexAop-rCD2::RFP, UAS-mCD8::GFP* (67093), *nompC-LexA* (52241), and *Gr64f-Gal4* (57668). The following lines were provided by the indicated investigators: *pain-Gal4*, *UAS-pain P103*, *UAS-pain P60* (gifts from W.D. Tracey) (3, 4), $pain^{pf}$ (Y.N. Jan)*, Gr66a-I-GFP, ppk28-LexA* (K. Scott) (5), *Gr64f-LexA, Gr66a-GAL4* (H. Amrein) (6), *ppk23-LexA* (B. Dickson), *Ir94e-LexA* (M. Stanley). We previously generated $trpA1^{1}$ (7) and $pain^{4}$ (8), which are available at the Bloomington Drosophila Stock Center (26504 and 600474, respectively). All *Drosophila* mutants were outcrossed to w^{1118} ≥5 generations. Because the mutants and transgenic lines harbor a w^{+} transgene, as the control for all experiments conducted in *Drosophila*, we used a modified w^{1118} background in which the X chromosome was replaced with that of Canton S (64349) yielding flies that are w^{+} on X, but the 2nd and 3rd chromosomes were derived from the w^{1118} strain.

Drosophila husbandry

Fruit flies were reared in vials or bottles containing standard cornmeal-yeast media at 25 °C in a 65% humidified chamber under 12 hr light/12 hr dark cycles. Crosses for the behavioral experiments were performed by placing 20–30 virgin females and 20–30 males in bottles. Other crosses were performed by placing 5–10 virgin females and 5–10 males in vials. Males and females were used equally in all experiments and were selected randomly. Following introduction in vials for egg laying, the adults were removed after 48-72 hours to limit population size, and promote health of the progeny.

Fruit flies were collected and aged for experiments as follows. For PER assays 10 males and 10 female flies were one-day post eclosion and aged in vials until 4-6 days old. Flies were starved for 20-22 hours at 25 °C under 60% humidity in vials that contained two Kimwipes saturated with 6 mL tap water. Experiments were performed 8-10 hours after light onset. For tip recording experiments, males and females were collected 0-1 days post eclosion, group housed in vials and aged for 5-10 days. Flies used for immunostaining and cDNA sample preparation were collected into vials one day post eclosion, group housed and aged until 5–7 days

old.

Mosquito stocks and husbandry

The Liverpool (LVP) strain of *Ae. aegypti* was used as the wild-type control in this study and was originally provided by O.S. Akbari (University of California, San Diego). All mutant mosquito lines were outcrossed to the LVP strain for ≥5 generations prior to experimentation. Mosquitoes were reared in walk-in environmental chambers maintained at 28 °C and 80% relative humidity under 14:10 h light:dark cycles in an ACL-2 laboratory. Eggs were hatched in either reverse osmosis or deionized water, and larvae were reared on fish food (TetraMin Tropical Granules, 16122, Tetra) until pupation. Pupae were transferred to insect collection cages (17.5 × 17.5 × 17.5 cm, BugDorm-4S1515) for adult emergence and maintenance. Adult mosquitoes were maintained on a 10% sucrose (w/v) solution provided via glass bottles with cotton wicks. For propagation and egg collection, females between 5 and ~30 days old were blood-fed using a membrane feeding system (SP6W1-3, HemoTek) containing defibrinated sheep blood (DSB250, HemoStat Laboratories, <7 days old).

cDNA synthesis and quantitative PCR (RT-qPCR)

Proboscises were dissected from 75 male and 75 female 5-7 day-old fruit flies. 60 proboscises, 60 forelegs, and 30 abdomens with the alimentary canal were dissected from 5-7-day-old female mosquitoes. RNAs were obtained using the RNAzol procedure (Molecular Research Center Inc., RN190) and cDNAs were generated using the reverse transcription system, iScript (Bio-Rad, 1708891). To perform RT-qPCR, we used the SYBR Green Super mix (Bio-Rad, 1708880). Relative RNA expression was determined using the $2^{-\Delta\Delta Ct}$ method (9) and adjusted to the level of expression of *RPS7*. The procedures were all performed in triplicate.

Generation of *Aedes* $\boldsymbol{pain1^{QF2}}$ and $\boldsymbol{pain1^{1}}$ mutants

The $pain1^{QF2}$ and $pain1^{1}$ alleles were generated using CRISPR-mediated homology-directed repair (HDR) as described previously with few changes (10). We selected short-guide RNAs (sgRNAs) that target *pain1* (AAEL006835-RA) using the CRISPR Optimal Target Finder (https://flycrispr.org/target-finder/). For the identification of transgenic mosquitoes, each allele carries a *3xP3*-driven fluorescent marker (DsRed for both $pain1^{QF2}$ and $pain1^{1}$).

To create the constructs for the CRISPR-mediated HDR, we first incorporated the sgRNAs into p*AaU6-LgRNA-T2A*::*QF2-3xP3-DsRed* (*SI Appendix* Fig. S9) or p*AaU6-LgRNA-3xP3-DsRed* (11). To do so we digested the plasmids with SpeI and KpnI and introduced the sgRNAs into the plasmids using the 5x In-Fusion Snap Assembly Master Mix (TaKaRa, ST2320). We amplified homology arms from the *pain1* gene using genomic DNA isolated from the LVP strain and the specified primer sets (*SI Appendix* Table 2). To complete the plasmid to create the $pain1^{QF2}$ allele, we introduced the 5' homology arm (1087 bp) into the AvrII site and the 3' homology arm (1086 bp) into the AfeI site of p*AaU6-LgRNA-T2A*::*QF2-3xP3-DsRed* with the sgRNA insertion. Similarly, to complete the plasmid to create the $pain1^{1}$ allele, we introduced the 5' homology arm (1000 bp) into the NheI site and the 3'

homology arm (1004 bp) into the PacI site of p*AaU6-LgRNA-3xP3-DsRed* with the sgRNA insertion. Both constructs, which had the *SV40* transcription terminator, sgRNA scaffold, *U6* promoter, and homology arms were verified by DNA sequencing.

To create the mutants, we microinjected at 400 ng/µL into embryos from a Cas9-expressing line (ubiquitin-L40 promoter, gift from O.S. Akbari) (12). G_0 survivors were self-crossed and the G_1 offspring were examined for successful insertions by examining the eyes for DsRed fluorescence, and then confirmed by RT-PCR (*SI Appendix* Fig. S4 *I-K*) as well as DNA sequencing (see *SI Appendix* Table 2 for sequencing primers). The confirmed mutants were outcrossed for 5 generations to the LVP strain and then homozygous lines were created.

Chemicals for tip recordings and behavioral assays

The following compounds were purchased from Sigma-Aldrich (St. Louis, MO): acetic acid (A6283), allyl isothiocyanate (AITC) (57067), butyric acid (B103500), caffeine (C8960), decanoic acid (C1875), DEET (317188-OFF), denatonium benzoate (D5765), ethyl alcohol (64175), glycolic acid (124737), HCl (A144-212), hexanoic acid (153745), lactic acid (L1750), linoleic acid (L1376), NaCl (S9888), quinine (Q1125), and sucrose (S0389). 50 mM stock solutions for denatonium, caffeine and quinine were dissolved in water before creating the working solutions used in the experiments. DEET was first dissolved in 100% ethanol, and then brought to working concentrations using MilliQ water. FAs were prepared as previously described (13). Short and medium chain FAs were first dissolved in 20% ethanol, brought to working concentrations using MilliQ water, and then sonicated to dissolve. Long chain FAs were first dissolved in 80% ethanol, brought to working concentrations, and then sonicated to dissolve. For flies experiments, all assays with short-, medium-, or long-chain FAs were performed with a final ethanol concentration of 9%. For mosquitoes, all assays with short- and medium-chain FAs used a final ethanol concentration of 2%, whereas assays with long-chain FAs used a final ethanol concentration of 8%. All control solutions contained matching ethanol concentrations to their corresponding test solutions.

Labellar proboscis extension response (PER) assays

5-7 day old *Drosophila* starved for 18-22 hr on water-soaked Kimwipes were used for labellar PER assays as previously described (14). Briefly, flies were immobilized in P20 tips such that only their heads with their labella were exposed, placed in a humidified chamber, and allowed to recover for 10-30 minutes. A small droplet of water was placed on the edge of the pipet tip within reach of their labella to prevent desiccation and to allow them to drink water *ad libitum* as they recovered.

Prior to performing the PER assays, the flies were satiated with water by touching their labella with a thin, cone-shaped Kimwipe wick soaked in water. Proboscis extension (extension of the proboscis accompanied by labial palp opening and closing) was scored as 1, and no response was scored as 0. When a fly responded (score = 1), it was allowed to drink ad libitum. Water stimulation and drinking were repeated until the fly no longer exhibited a proboscis extension to

water. Flies that drank for longer than 2 minutes total or continued to respond to water for more than 2 minutes were discarded.

Following satiation, each fly was tested with 100 mM sucrose as a positive control. Flies that failed to respond to sucrose were excluded. Flies were then tested with water as a negative control. In the rare instance that a fly responded to the negative control (indicating incomplete satiation), the satiation procedure was repeated and both controls were reassessed before proceeding with the assay.

Prior to stimulation with a wick soaked in a compound of interest, flies were stimulated with water to ensure that the following trial was due to the tested compound rather than thirst. In the rare occasion that a fly responded to the water stimulation, the fly was satiated and the trial was repeated. If the fly continued to respond to water after one repeat, the fly was discarded. Following each trial, a droplet of water was placed at the edge of the pipet tip to prevent desiccation between trials. Flies were tested with 30 mM sucrose with the appropriate solvent for the assay (9% ethanol for FAs, MilliQ water for other compounds such as denatonium), and then 30 mM sucrose plus the taste compound of interest. Following the assay, flies were tested with 100 mM sucrose to ensure they did not die or lose labellar sensitivity during the assay.

Tarsal proboscis extension response (PER) assays

5-7-day-old *Drosophila* starved for 18-22 hours on water-soaked Kimwipes were prepared for tarsal PER assays after brief anesthetization on ice (<2 minutes). Flies were immobilized on a cover slip by gently positioning each fly's dorsal thorax on a small droplet of clear nail polish. Flies were allowed to recover for >30 minutes in a humidified chamber at 25 °C.

Following recovery, flies were satiated with water by touching their forelegs and labella with water-soaked Kimwipes until they no longer responded to a brief water stimulation. Proboscis extensions in response to stimulation with a small <10 µL droplet of tested compound (proboscis extends and labial palps flap) were recorded as 1, and no response was recorded as 0. Flies that continued to respond to water or drank water for more than 2 minutes were discarded. As a positive control, the first tarsal segments on the forelegs of each fly were stimulated with 100 mM sucrose. Flies that did not respond to sucrose were discarded.

Prior to tarsal stimulation with the tested concentration of FA mixed with 30 mM sucrose, flies were stimulated with water to ensure that the following trial was due to the tested compound rather than thirst. In the rare occasion that a fly responded to the water stimulation, the fly was satiated and the trial was repeated. If the fly continued to respond to water after one repeat, the fly was discarded. To conclude each experiment, the tarsi were stimulated with 100 mM sucrose to ensure that the fly was still alive and did not lose tarsal sensitivity.

Blood feeding assays

To conduct assays with either a single blood feeder or with two blood feeders (two-way choice assay), 50 adult, mated, female *Ae. aegypti* (5-10 days old) were

selected and kept in a cage (17.5 × 17.5 × 17.5 cm, BugDorm-4S1515) with 10% sucrose for ≥24 hours in a walk-in chamber maintained at 28 °C and 80% relative humidity. VectaDerm (a skin-like artificial membrane) was soaked in MilliQ water plus the solvent (8% ethanol) or water containing the indicated concentrations of FAs plus the solvent in a Petri dish for ≥45 minutes. The wet membranes were set on the prewarmed feeders (Hemotek PS6 Membrane Feeding System). For the single blood feeder assays, each feeder was filled with defibrinated sheep blood (HemoStat Laboratories) until there was no gap between feeder and membrane and allowed to warm to 32-33 °C.

For the two-way choice assays, the two blood feeders were placed 4-5 cm apart. The blood in one feeder had 0.1% fluorescein dye (Sigma, 518-47-8) and the other blood option had 0.1% rhodamine B dye (Thermo Fisher Scientific, 81-88-9). One of the two blood feeders had the VectaDerm soaked in water plus solvent (8% ethanol), while the other blood feeder had the VectaDerm soaked in the indicated percentage of FAs plus the solvent. We switched the addition of the fluorescein and rhodamine dyes in every set of experiments to ensure that the dyes did not influence the outcomes. We gently introduced the feeders inside the cage with the 50 female mosquitoes, making sure that chemicals on the membranes did not touch the mesh of the cage. In some experiments, both blood feeders were exposed to 5% CO_2, which was delivered through a perforated tube introduced inside the cage by placing an opening of CO_2 tubes between the blood feeders. In other experiments, the blood feeders exposed to CO_2 were also exposed to human odor by placing a worn nitrile glove inside the cage between the two blood feeders.

The females were allowed to blood feed for 10 minutes before we transferred the cages to a -20 °C freezer for ≥ 1 hour. For the single blood feeder assays, we counted the number of engorged females, and calculated the percentages that engorged. For the two-way choice assays, we examined the abdominal colors using a Zeiss SteREO Microscope (Discovery. V8; S/N 2852) with 488 nm (GFP, green) and 530 nm (rhodamine, red) channels. Mosquitoes were scored as having abdomens that were red (R), green (G), or that were both red and green (B). Preference indexes (PIs) were calculated as follows: $PI=(N_G-N_R)/(N_G+N_B+N_R)$ or $(N_R-N_G)/(N_R+N_B+N_G)$.

Landing assays

We identified mosquitoes that landed on a blood feeder by the presence of rhodamine B on their legs and labellum. To conduct this analysis, we soaked the VectaDerm in 8% ethanol and 0.1% rhodamine B (control) or 1% LA with 8% ethanol and 0.1% rhodamine B. The membrane was mounted on a Hemotek blood feeder filled with defibrinated sheep blood. 50 female mosquitoes were allowed to interact with the blood feeder for 10 minutes and frozen at -20 °C for ≥ 1 hour. To score landings, we examined the deposition of fluorescent dye on the tarsi and the tip of the labellum using a Zeiss SteREO Microscope (Discovery. V8; S/N 2852) with the 530 nm (rhodamine, red) channel.

Nectar feeding assays

50 adult female mosquitoes (4-10 days old) were selected and kept in a cage (17.5 × 17.5 × 17.5 cm, BugDorm-4S1515) for 36 hours with water only in a walk-in chamber held at 28 °C and 80% relative humidity. We prepared two food options– one with 20 mM sucrose solution and either sulforhodamine (0.09 mg/mL) or brilliant blue FCF dye (0.09 mg/mL), and the other with 20 mM sucrose mixed with the indicated concentration of FAs plus solvent (8% ethanol) and either sulforhodamine or brilliant blue FCF dye, so that the two options had different dyes. We dispensed the two food options into 96-well plates (Corning® 96 Well Clear Polystyrene Microplate; CLS3795) in alternating wells (*SI Appendix* Fig. S5*G*). Half the wells were kept empty. We introduced the plates into cages and allowed the females to feed for 3 hours, transferred the cages to a -20 °C freezer for ≥1 hour, and scored the abdominal colors: blue (B), red (R), and purple (P). Preference indexes (PIs) were calculated as follows: $PI=(N_B-N_R)/(N_B+N_P+N_R)$ or $(N_R-N_B)/(N_R+N_P+N_B)$.

Olfactory assays using a Y-tube olfactometer

To assay olfactory behavior, a custom-built Y-tube olfactometer was constructed by the UCSB Physics Machine Workshop (https://www.physics.ucsb.edu/pro-machine-shop), following a previously reported design (1) with minor modifications (*SI Appendix* Fig. S6*A*). The main body consisted of a large polycarbonate tube (45 cm long × 8 cm diameter), connected on one end to a mosquito holding chamber (24 cm long × 8 cm diameter) and on the other end to two lateral arms (each 30 cm long × 8 cm diameter), which served as traps. Each trap was further connected to a sample-holding chamber (10 cm long × 8 cm diameter) via a mesh screen to prevent direct contact between the stimulus and the mosquitoes. Tubing delivering 5% CO_2 was connected to each arm.

The behavioral assays were conducted in a walk-in chamber maintained at 28 °C and 80% relative humidity. To minimize external visual cues and to simulate dawn/dusk conditions preferred by host-seeking mosquitoes, the area used for the assays was enclosed with black curtains so that illumination was kept low (~10 lux). For each trial, 20 mated, 7–10-day-old female *Aedes aegypti* were introduced into the holding chamber and allowed to acclimate for 10 minutes before being released. During the 10 minute acclimation period, the stimulus and control solutions were prepared by pipetting 20 mL of the test or the control solution into 2-oz plastic cups (Karat FP-P200-PP), each containing a sterile cotton ball to prevent spillage and ensure even vapor distribution. The cups were placed inside the sample-holding chambers.

To initiate the assays, a 5% CO_2 source was turned on, the butterfly valve was rotated to allow mosquitoes to enter the olfactometer and choose between the two arms for 10 minutes. At the end of each trial, the numbers of mosquitoes present in the test and reference (control) arms were counted. Individuals that remained in the holding chamber or did not enter either trap were excluded from the analysis. We only included experiments in which there was ≥80% participation (≥16 mosquitoes). ~90% of experiments were equal to or exceeded this threshold. Preference indexes (PIs) were calculated as follows where N_{test} is the side with CO_2 and FAs and N_{ref} is the side with CO_2 only: $PI=(N_{test}-N_{ref})/(N_{test}+N_{ref})$. To prevent positional bias, the test and control stimuli were alternated between the two arms across replicates. The

apparatus was cleaned with 70% ethanol and allowed to dry between trials to remove residual odors.

Immunostaining

Fruit flies: Labella and tarsi from 5–7-day-old flies were dissected at room temperature in chilled PBST0.3 (PBS and 0.3% Triton X-100). Tissues were fixed in 4% paraformaldehyde in PBST0.3 for 1 hour at 4 °C. Samples were washed in PBST0.3 (15-20 min, three times) and blocked with 5% normal goat serum (MP Biomedicals) in PBST0.3 (block buffer) for 30 minutes at room temperature while rotating, followed by incubation with primary antibodies in blocking buffer overnight at 4 °C while rotating. The samples were washed with PBST0.3 (10 minutes, three times) and incubated with the secondary antibodies in a blocking buffer overnight at 4 °C in the dark. After a final PBST0.3 wash (10 minutes, three times), the samples were mounted using Vectashield Antifade Mounting Media (Vector Laboratory Inc., H-1000) and 1.5H high performance coverslips (Carl Zeiss™ 474030-9000-000), and were secured with nail polish. The samples were imaged using a Zeiss LSM 900 confocal laser scanning microscope using a 20x/0.8 Plan-Apochromat DIC objective. The images were processed using Zen Blue software and ImageJ. The following primary and secondary antibodies were used at the indicated dilutions: chicken anti-GFP (1:1000; Thermo Fisher Scientific), rabbit anti-DsRed (1:500; Clontech), AlexaFluor 488 goat anti-chicken IgG (1:1000; Thermo Fisher Scientific), and AlexaFluor 568 goat anti-rabbit IgG (1:1000; Thermo Fisher Scientific).

Mosquitoes: 5–10-day-old female mosquitoes were anesthetized on ice, and the labella and tarsi from forelegs were dissected with fine forceps under a stereomicroscope. The tissues were fixed at 4 °C for 4 hours in 4% paraformaldehyde in PBST1 (PBS and 1% Triton X-100) in 1.5 mL microcentrifuge tubes. To make sure that all samples were fixed, we verified that the samples completely sank into the fixative solutions. The samples were washed three times (15 minutes each) with PBST1 on a low-speed horizontal shaker. The tissues were permeabilized by incubating in Intercept (PBS) blocking buffer (LI-COR, Part No. 927-70001) overnight (~12 hours) at 4 °C. Following permeabilization, the solutions were replaced with fresh blocking buffer, and the samples were incubated with primary antibodies overnight at 4 °C. The primary antibodies were mouse anti-GFP (1:1000; A11120, Invitrogen) to identify mCD8::GFP reporter expression. The samples were washed three times with washing buffer for 15 minutes each at 4 °C, incubated with secondary antibodies (goat anti-mouse Alexa Fluor 488; 1:500; Life Technologies A-11001) in Intercept Blocking Buffer with PBS for 12 hrs at 4 °C, and then washed four times with a PBST1 for 15-minutes each. The samples were mounted on glass slides with Vectashield Antifade Mounting Media (Vector Laboratories Inc., H-1000), incubated overnight at 4 °C, and viewed using a Zeiss confocal microscope (LSM 900). Final image processing was done with ImageJ software.

Scanning electron microscopy

Mosquitoes were immobilized on Petri dishes, which were placed on ice, and their heads were dissected at the base immediately before imaging. The heads were

placed on double-sided adhesive tape, and tiny strands of tape were applied across the proboscises. Specimens were arranged in different orientations to view the labella from either the ventral or dorsal perspectives, and images were captured with a Thermo Fisher Scientific ApreoS 13.5.0 (serial number 9950439; Materials Department Microscopy Core, UCSB).

Assaying tastant-induced action potentials by performing tip recordings from gustatory sensilla on the labella

Fruit fly sample preparation: Tip recordings were performed on 5-10-day-old fruit flies as previously described (15). For most experiments, the genotypes of the flies were blinded by the experimenter performing the recordings.

Mosquito sample preparation: Tip recordings were conducted on female mosquitoes 5–10 days post eclosion. Mosquitoes were cold-anesthetized on ice, and all six legs were gently removed using fine forceps. The bodies were positioned with the ventral side down on a square piece of double-sided adhesive tape such that only the distal tips of the labellar palps extended beyond the edge of the tape. Four additional narrow strips of double-sided tape were applied over the thorax, base of the labium, base of the labellum, and the distal abdomen to ensure stable fixation throughout the recordings. A reference electrode filled with Ringer's solution (16) was inserted into the thorax.

Tip recordings: All chemicals were dissolved in 30 mM TCC, which served as the electrolyte. The solutions were backfilled into the recording electrodes (World Precision Instruments, 1B150F-3) with 20 μm openings (Sutter Instrument, P-97 puller). Due to the high concentrations of the FAs tested on *Drosophila*, to perform the tip recordings, the FAs were dissolved in 30 mM TCC and 9% ethanol. To perform the tip recordings with *Ae. aegypti*, linoleic acid was dissolved in 30 mM TCC and 8% ethanol, and the hexanoic acid was dissolved in 30 mM TCC and 2% ethanol. The electrodes were inserted over the indicated sensilla and signals were amplified and digitized with an IDAC-4 data acquisition device and Autospike software (Syntech Oeckenfels GmbH). The electrical signals were amplified (10×) using a Syntech signal connection interface box with a 100–5000 Hz band-pass filter. The spikes were digitized at a sampling rate of 12 kHz and analyzed with Autospike 3.1 (Syntech Oeckenfels GmbH). All tip recordings were performed for 10 seconds. Spike sorting was used to identify spike amplitudes that correspond to the action potentials of sweet and bitter-responsive GRNs. Tastant-induced spikes were quantified by counting the number of action potentials for 1 second for flies and 500 ms for mosquitoes, excluding the first 50 ms, which often includes a contact artifact. Each compound was tested on ≥8 biological replicates, with one insect used per replicate.

Electroantennogram (EAG) recordings

EAG recordings on female mosquitoes (aged 6–8 days) were performed as previously described (17) with minor modifications. The mosquitoes were briefly anesthetized on ice and transferred to a cold platform (Model 1429 Chill Table, BioQuip) for dissection. The distal tips of both antennae were trimmed using fine

scissors under a stereomicroscope (AmScope SW-2B13-6WA-V331), and the remaining antennae were gently dipped in electrode gel (Spectra 360, Parker Laboratories) to adhere them together for enhanced signal quality. The heads were then excised and positioned on a reference electrode consisting of a chlorinated silver wire (World Precision Instruments) inserted into a borosilicate glass capillary (1B150F-3, World Precision Instruments) pulled using a P-97 puller (Sutter Instruments). The reference electrode was filled with saline solution containing 150 mM NaCl, 25 mM HEPES, 5 mM glucose, 3.4 mM KCl, 1.8 mM $NaHCO_3$, 1.7 mM $CaCl_2$, and 1 mM $MgCl_2$. The pH was adjusted to 7.1 using 1 M NaOH.

To perform each recording, a mounted head was transferred to the EAG setup, and the tips of both antennae were inserted into a recording electrode, also containing the same saline solution. A steady stream of humidified air was applied from a ~1 cm distance from the antennae using a glass Pasteur pipet (Stimulus Controller CS 55, Syntech Oeckenfels GmbH). 40 µL of odorant was applied to a small, round filter paper and placed inside a glass Pasteur pipet, which was then introduced into the continuous air stream through a small hole to allow the molecules to mix with clean, humidified air. The stimuli were applied as a 1-second pulse with a 30-second recovery period. The responses were amplified and recorded using an IDAC-4 data acquisition system (Syntech Oeckenfels GmbH) with EAGpro software (Syntech Oeckenfels GmbH) at the rate of 5 kHz. 1% hexanoic acid was prepared in unscented paraffin oil (Patterson Medical), and unscented paraffin oil was used as vehicle control.

Cell culture, molecular cloning, and transfections for Ca^{2+} photometry

HEK293 cells were maintained in high-glucose DMEM (Gibco) supplemented with 10% heat-inactivated fetal bovine serum (GenClone) and 1% Penicillin-Streptomycin (Invitrogen) at 37 °C in a humidified incubator with 5% CO_2. Cells were seeded onto poly-L-lysine–coated 12 mm glass coverslips (Warner Instruments) in 12-well plates (Thermo Fisher Scientific, 150628) 24 hours before transfection.

The *Drosophila melanogaster pain* plasmid (pCMV-*dPain*) was used as previously described (18). The *Aedes aegypti pain1* (*Aapain1*) gene (2742 bp) was synthesized as a codon-optimized gene block (IDT) based on the reference sequence XM_001652211. The pcDNA3.1 vector was linearized using NheI (New England BioLabs), and the *pain1* insert was cloned into the vector using In-Fusion Snap Assembly (Takara Bio). The final construct was verified by DNA sequencing using Oxford Nanopore plasmid sequencing (Plasmidsaurus).

HEK293 cells were transfected with 1 µg of either pCMV-*dPain* or pcDNA3.1-*Aapain1*, along with 0.1 µg of p*cDNA3-DSRed-Express2* (Addgene plasmid #128040; a gift from Oskar Laur), using XtremeGENE 9 transfection reagent (Roche) according to the manufacturer's protocol. After transfection, cells were incubated at 37 °C for 3–4 hours. To avoid thermal activation of the channels, the cells were transferred to a 33 °C incubator for 36 hours prior to imaging as described (18).

Ca^{2+} photometry

36–40 hours post-transfection, HEK293 cells were incubated with 5 µM Fluo-8 AM dye (AAT Bioquest) in imaging buffer (in mM: 140 NaCl, 5 KCl, 2 $MgCl_2$, 2 $CaCl_2$, 10 HEPES, and 10 glucose, adjusted to pH 7.4 with NaOH) containing 0.04% Pluronic® F-127 (Sigma-Aldrich) and 1 mM probenecid (Sigma-Aldrich) at 33 °C for 1 hour. The same buffer was used for all imaging and perfusion experiments. Following dye loading, the coverslips were mounted in a recording chamber (RC-25F; Warner Instruments) and connected to a gravity-driven perfusion system. Cells were illuminated using a xenon arc lamp (Lambda LS; Sutter Instruments), and fluorescence was excited at 488 nm and collected at 510 nm using a Moment CMOS camera (Teledyne Vision Solutions). Fluorescence intensity was acquired and analyzed using NIS-Elements AR software (Nikon Instruments). For chemical stimulation, 100 µM linoleic acid was added to the bath solution from a 10% stock solution prepared in 80% ethanol. AITC was prepared from 1 M stock in 100% DMSO and then diluted to 1 mM in recording solution. Thermal stimulation was applied using a pre-heated buffer delivered via an in-line solution heater (SH-27B; Warner Instruments). Each condition was tested in three independent experiments. The changes in fluorescence from ~10 cells/experiment (n ≈ 30 total) were quantified.

Quantification and statistical analysis

All error bars represent standard error of the means (SEMs). All data were plotted using Prism 10 software (GraphPad). The number of times each experiment was repeated (n) are indicated in the figure legends. For assays where multiple individuals are represented within a single ‘n’, we indicate the number of individual insects tested per ‘n’ with ‘N’. For imaging assays, each ‘n’ represents an individual whole-mounted tissue. For the PER assays each ‘n’ represents an experiment performed on a single day with 10-12 fruit flies per genotype repeated over 5 independent days. Each ‘n’ for the tip recordings represents the firing rate for a single indicated sensillum. For the tip recordings, we estimated the sample size using preliminary data obtained with control and *pain* or *pain1* mutant insects with an n=3. We set the significance level, $\alpha=0.05$ and power, $1-\beta=0.9$. For mosquito behavioral assays and Ca^{2+} photometry we used the standard for our lab to determine ‘n’s.

The Mann-Whitney U test was applied for two-group comparisons (i.e., the difference between controls and mutants). Differences among multiple groups (i.e., for testing concentration dependence in blood feeding assays within genotypes where separate groups are compared to the same control) were analyzed by one-way ANOVA followed by Dunnett’s post-hoc test. Residuals were tested for normality using the Shapiro-Wilk test and for equal variances with the Brown-Forsythe test. For datasets that violated parametric assumptions, the Kruskal–Wallis test with Dunn’s multiple-comparison adjustment was performed for multi-group analyses. Paired datasets generated with the PER assays in Fig. 2, Fig. 3, and *SI Appendix* Fig. S2 *A* and *B* were analyzed using repeated-measures one-way ANOVA with the Geisser-Greenhouse correction. Residuals were checked for normality using the Shapiro-Wilk test and sphericity (equal variability) was not assumed. If the Geisser-Greenhouse $\boldsymbol{\varepsilon}<0.75$, the corrected p-values were used. We

used paired Student's *t*-tests to compare the frequencies of action potentials between the first versus either the second or third stimulations with the same chemical (*SI Appendix* Fig. S7*G*). The statistical tests applied are noted in each figure legend. All analyses were performed in GraphPad Prism 10. Statistical significance is denoted by asterisks: One asterisk, $P < 0.05$. Two asterisks, $P < 0.01$. Three asterisks, $P < 0.001$.

Supplementary references

1. Rodriguez SD, Drake LL, Price DP, Hammond JI, & Hansen IA (2015) The efficacy of some commercially available insect repellents for *Aedes aegypti* (Diptera: Culicidae) and *Aedes albopictus* (Diptera: Culicidae). *J. Insect Sci.* 15:140.
2. Storelli G*, et al.* (2011) Lactobacillus plantarum promotes *Drosophila* systemic growth by modulating hormonal signals through TOR-dependent nutrient sensing. *Cell Metab.* 14:403–414.
3. Tracey WD, Wilson RI, Laurent G, & Benzer S (2003) *painless*, a *Drosophila* gene essential for nociception. *Cell* 113:261–273.
4. Hwang RY, Stearns NA, & Tracey WD (2012) The ankyrin repeat domain of the TRPA protein Painless is important for thermal nociception but not mechanical nociception. *PLoS One* 7:e30090.
5. Wang Z, Singhvi A, Kong P, & Scott K (2004) Taste representations in the *Drosophila* brain. *Cell* 117:981–991.
6. Dunipace L, Meister S, McNealy C, & Amrein H (2001) Spatially restricted expression of candidate taste receptors in the *Drosophila* gustatory system. *Curr. Biol.* 11:822–835.
7. Kwon Y, Shim HS, Wang X, & Montell C (2008) Control of thermotactic behavior via coupling of a TRP channel to a phospholipase C signaling cascade. *Nat. Neurosci.* 11:871–873.
8. Liu J*, et al.* (2023) Alleviation of thermal nociception depends on heat-sensitive neurons and a TRP channel in the brain. *Curr. Biol.* 33:2397–2406 e2396.
9. Livak KJ & Schmittgen TD (2001) Analysis of relative gene expression data using real-time quantitative PCR and the $2^{-\Delta\Delta C_T}$ Method. *Methods* 25:402–408.
10. Meyerhof GT*, et al.* (2025) Visual threat avoidance while host seeking by *Aedes aegypti* mosquitoes. *Cell Rep.* 44:115435.
11. Wang Y*, et al.* (2024) Deafness due to loss of a TRPV channel eliminates mating behavior in *Aedes aegypti* males. *Proc. Natl. Acad. Sci. U.S.A.* 121:e240432412.
12. Li M*, et al.* (2017) Germline Cas9 expression yields highly efficient genome engineering in a major worldwide disease vector, *Aedes aegypti*. *Proc. Natl. Acad. Sci. U.S.A.* 114:E10540–E10549.
13. Ahn JE, Chen Y, & Amrein H (2017) Molecular basis of fatty acid taste in *Drosophila*. *eLife* 6:e30115.
14. Shiraiwa T & Carlson JR (2007) Proboscis extension response (PER) assay in Drosophila. *J. Vis. Exp.* 193:doi: 10.3791/3193.
15. Delventhal R, Kiely A, & Carlson JR (2014) Electrophysiological recording from *Drosophila* labellar taste sensilla. *J. Vis. Exp.*:e51355.
16. Dhakal S, Sang J, Aryal B, & Lee Y (2021) Ionotropic receptors mediate nitrogenous waste avoidance in *Drosophila melanogaster*. *Commun Biol* 4:1281.
17. Lahondere C (2021) A step-by-step guide to mosquito electroantennography. *J. Vis. Exp.* 10 10.3791/62042.
18. Sokabe T, Tsujiuchi S, Kadowaki T, & Tominaga M (2008) *Drosophila* Painless is a Ca^{2+}-requiring channel activated by noxious heat. *J. Neurosci.* 28:9929–9938.
